# Antidepressant use in spatial social networks

**DOI:** 10.1126/sciadv.adr0302

**Published:** 2024-12-06

**Authors:** Balázs Lengyel, Gergő Tóth, Nicholas A. Christakis, Anikó Bíró

**Affiliations:** ^1^ MTA–HUN-REN “Momentum” Agglomeration, Networks, and Innovation Research Group, HUN-REN Centre for Economic and Regional Studies, 1097 Budapest, Hungary.; ^2^ ANETI Lab, Corvinus Institute for Advanced Studies, Corvinus University of Budapest, 1093 Budapest, Hungary.; ^3^ Institute of Data Analytics and Information Systems, Corvinus University of Budapest, 1093 Budapest, Hungary.; ^4^ CERUM, Umea University, 901 87 Umea, Sweden.; ^5^ Human Nature Lab, Yale University, New Haven, CT 06520-8263, USA.; ^6^ Health and Population Research Group, HUN-REN Centre for Economic and Regional Studies, 1097 Budapest, Hungary.

## Abstract

Social networks may help individuals maintain their mental health. Most empirical work based on small-scale surveys finds that cohesive social networks are critical for mental well-being, while diverse networks are considered less important. Here, we link data on antidepressant use of 277,344 small-town residents to a nationwide online social network. The data enable us to examine how individuals’ mental health care is related to the spatial characteristics of their social networks including their ties in the local community and connections to distant communities. We find that, besides the cohesion of social networks around home, the diversity of connections to distant places is negatively correlated with the probability of antidepressant use. Spatial diversity of social networks is also associated with decreasing dosage in subsequent years. This relationship is independent from the local access to antidepressants and is more prevalent for young individuals. Structural features of spatial social networks are prospectively associated with depression treatment.

## Teaser

Antidepressant purchase data linked to an online social network reveal the role of diverse networks in mental health.

## INTRODUCTION

The theory of social capital (*1*, *2*) has been used to help understand mental health inequalities (*3*, *4*). Relatedly, social support interventions have become part of health policy directed at mental disorders (*5*). A rich literature suggests that social connections (the structural form of social capital) can reduce stress, anxiety, and depression (*6*–*11*). The central tenet is that mental balance can be primarily maintained with the help of emotional support gained from cohesive networks where individuals can access “bonding” social capital and ask help from their strong ties (*12*). Fewer investigators have argued for or explored the importance of diverse networks that can mobilize “bridging” social capital (*13*, *14*), despite their pivotal role in providing, say, economic opportunities (*15*–*17*) that subsequently influence health outcomes (*18*). A potential reason for the paucity of investigations of this kind is that previous empirical work has mostly used small-scale surveys to collect social connections that could reveal the strength of dyadic relationships in depth (*8*, *10*) and could capture the size of ego networks (*19*) but had limitations in mapping triadic closure of friendship at scale (*20*). These data limitations have made the mental health outcomes of cohesive versus diverse network structures, and thus of bonding versus bridging social capital, difficult to compare.

Here, we examine a nationwide dataset on antidepressant use linked to the online social network (OSN) of 277,344 small-town residents; this provides us an informative scale for the social network analysis of mental health. By using network structure measures, our data enable us to demonstrate the importance of both network cohesion and diversity in mental health. However, disentangling bridging and bonding within and across groups remains difficult without considering the characteristics of individuals and homophilia in their relationships (*2*). In that regard, we exploit the geography of the network and analyze cohesive and diverse relationships within and across towns. Co-location of individuals in towns and in neighborhoods has a strong impact on their social capital (*17*) and can capture bonding mechanisms of social support and control (*21*). Furthermore, social capital falls with distance due to higher establishment and maintenance costs (*22*, *23*) and it is an empirical fact that the probability of links and network cohesion decreases with geographic distance (*24*–*26*), all suggesting that geographically remote connections in OSNs are likely weak despite the relatively rare strong links that do bridge distant places (*27*). The spatial diversity (SD) of social connections correlates with individual economic wealth, indicating that links across towns can be associated with bridging social capital (*16*). We proxy bonding social capital with a local cohesion (LC) indicator, which captures the tendency to participate in cohesive networks in subjects’ hometowns, and we define an SD index that captures the capacity to connect diverse communities in distant towns as a proxy for bridging social capital.

We find that LC as well as SD predict the probability of antidepressant use with negative coefficients and confirm that both bonding and bridging social capital are related to mental health. However, the significance of LC as a predictor of antidepressant use vanishes at high levels of SD, suggesting that links to distant communities can compensate for the lack of bonding social capital in the local environment. Furthermore, the dosage of antidepressants in subsequent years decreased more for patients who have spatially diverse networks. This latter relationship is stable after controlling for the local accessibility of antidepressants and is stronger for young individuals than for the elderly. Overall, the results suggest that diverse social networks with distant friends have a more important role in mental health care than previously thought.

## RESULTS

### Data

**Fig. 1.**
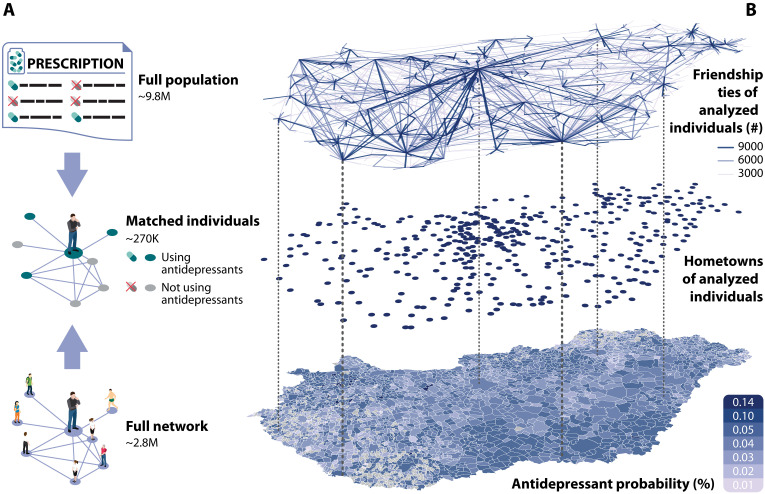
Preparation and geographies of the data. (**A**) A nationwide dataset of antidepressant prescriptions is linked to the OSN of small-town residents in Hungary at the individual level. (**B**) Hometowns of analyzed individuals and geographies of their social networks and local ratio of antidepressant users.

We link antidepressant use to an OSN at the individual level (Fig. 1). Antidepressant use is retrieved from prescriptions that cover the entire population of Hungary in the 2011 to 2015 period, and we also observe the dosage of antidepressants that the patients purchased (described in Materials and Methods and in section S1). This prescription data do not capture undiagnosed mental problems or treatments without medication, but it does indicate depression, anxiety, or sleeping disorders (*28*–*31*) on a population scale. The OSN data come from the International Who Is Who (iWiW) social media portal that counted for 30% of the country’s total population in 2011. The iWiW data are a collection of friends, including their self-reported place of residency (*26*); these data have been previously used to measure the relationship of network structural characteristics of bonding and bridging social capital with corruption (*32*) and income inequality (*33*). Our data are similar to other OSN sources that measure social capital at scale (*17*, *34*). Actual postings were rare on iWiW because it mainly functioned as a phone book and so was not very likely to induce anxiety like forms of social media that emerged later (*35*–*37*). Because of low costs of tie maintenance, distance decay in this network (*26*) is smoother than reported earlier for phone calls (*25*). Although tie strength cannot be measured, the average degree (〈k〉≅200) is larger than proposed by the social brain hypothesis on a manageable ego-network size (*19*) that signals the presence of strong and weak ties as well.

We restrict the prescription dataset to all residents of 409 Hungarian small towns with a population size between 5000 and 20,000, which enables the probabilistic matching with the OSN data. Estimated false matching probability remains under 4% in this town sample. We describe the ethical permissions and the matching process in the Data and Methods sections and provide further details on matching quality and potential sources of noise in section S2.

The resulting dataset contains 277,344 individuals with zero or positive prescription antidepressant use over the 2011 to 2015 period and network characteristics for the year 2011 (Fig. 1A). These analyzed individuals live in towns both of high and low ratio of antidepressant users and are distributed across the country (Fig. 1B). The spatial structure of the analyzed social networks resembles the geographies of the entire iWiW population as previously documented (*25*). A town-level analysis described in detail in section S3 confirms that the sample towns have higher rates of iWiW users than nonsample towns, suggesting good network representation, but they do not differ significantly from the rest of the country in terms of the fraction of antidepressant users (two-sided Mann-Whitney *U* test, *P* = 0.971). However, the fact that the prevalence of depressive symptoms in Hungary is high (*38*) while the prevalence antidepressant use is around the EU average (*39*) or lower (*40*) suggests that antidepressant usage does not entirely cover depressive symptoms in our case. Remaining data limitations are detailed in Discussion.

### Variables

Antidepressant use is captured by the binary variable *A_i_* ∈ (0, 1) (*N* = 277,344) that takes the value of 1 if individual *i* purchased at least one package of antidepressants prescribed by a practitioner in 2011. On the town level, high values of the mean of *A_i_* are clustered in space (Fig. 1B) and are positively correlated with log-transformed town population in a univariate linear regression (β = 0.038, *P* = 0.041) and negatively with average income (log-transformed, β = −0.198, *P* = 8.84 × 10^−8^) (see section S3). A total of 10,291 individuals (3.7%) have taken antidepressants in our small-town sample in 2011.

We define the network cohesion and diversity by quantifying the structure of ego networks constructed from the full iWiW network. That is, we include all iWiW users living in all Hungarian towns to construct ego networks of analyzed individuals *i* in the restricted sample described above. Testing the correlation between network cohesion measured by the clustering coefficient and antidepressant use, we find a reversed U-shape. This result signals that both network cohesion and diversity can be important for mental health (see section S4 for details). Then, we exploit the geographical dimension of the network to disentangle cohesion from diversity and investigate network structures within and across towns. Detailed description and illustration of our final variables can be found in section S5.

**Fig. 2.**
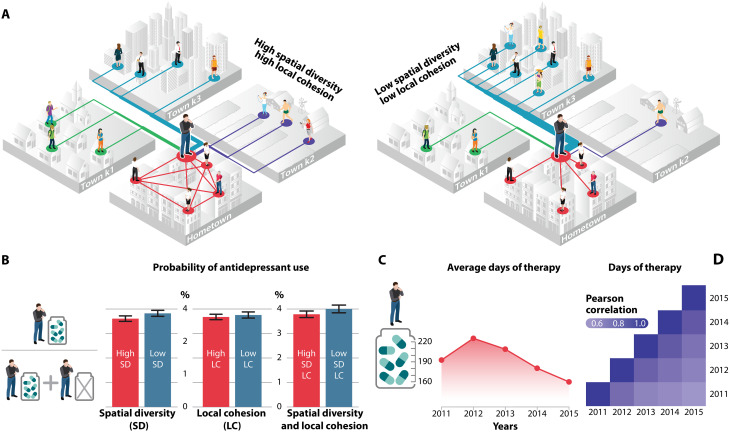
Spatial social network variables. (**A**) Ego’s network structure is characterized by the LC of social network within ego’s town and by the SD of connections across towns. (**B**) The probability of taking antidepressants is lower for individuals who have above-median SD. (**C**) The average dosage of antidepressants in our patient sample is decreasing over the observed period. (**D**) The decreasing correlation over the years signals that individual dosage is changing.

The local cohesion of social ties LC*_i_* is defined as a function of network clustering and is used as a proxy for the spatial dimension of bonding social capital. LC*_i_* measures the degree to which the *j* and *k* connections of individual *i* residing in *i*’s hometown *h* are linked to each other

$${\text{LC}}_{i}=\frac{2L_{\mathrm{jk}}\in h}{n_{i}\in h\left[\left(n_{i}\in h\right)-1\right]}/C_{i}^{\text{ER}}$$ (1)

where *L_jk_* ∈ *h* is the number of links among *n_i_* ∈ *h* connections of *i* (*j*, *k* ∈ *n_i_*). We normalize the observed clustering of *i* by the average clustering in *i*’s ego network in 10 simulated Erdős-Rényi (ER) random networks of *n_i_* nodes and *L_jk_* connections (*C_i_*^ER^), in which clustering is independent of degree (*41*) but edges in the ego network can be reshuffled within and across town borders as well. Higher values of LC*_i_* imply that *i* belongs to a strongly knit local community compared to spatially randomized networks of identical size, while low values imply that *i* does not have a cohesive social network in her hometown (Fig. 2A). Comparing observed network clustering within the town to ER random networks helps us overcome the complex problem that local and nonlocal triadic closure can be interrelated.

Following (*16*), we proxy spatial bridging social capital by spatial diversity SD*_i_* in the ego network of individual *i* using the Shannon entropy of connections across towns that we normalize by its theoretical maximum

$${\text{SD}}_{i}=\frac{-\sum_{g=1}^{G}p_{\mathrm{ig}}\text{ln}\left(p_{\mathrm{ig}}\right)}{\text{ln}\left(G_{i}\right)}$$ (2)

where *G_i_* is the number of different towns where connections of *i* live (*h_i_* ∈ *G_i_*), *p_ig_* is the proportion of the total number of *i* connections to town *g*. $p_{\mathrm{ig}}=L_{\mathrm{ig}}/\sum L_{i}$, where *L_ig_* is the number of social ties of *i* to town *g*, and the denominator is the total number of friendships of *i*. High SD_*i*_ implies that the individual splits their social connections more evenly among other towns, while low values imply that the individual’s ties are concentrated in a relatively small number of towns (Fig. 2A).

The probability of using antidepressants is slightly lower for individuals who have above-medium LC*_i_* and is significantly lower for those individuals who have above-medium SD*_i_* (Fig. 2B). Next, we quantify the days of therapy (DOT) of those patients who take antidepressants in 2011. *Z_i,t_* measures DOT for each antidepressant type by multiplying the volume of antidepressant packages purchased in year *t* by individual *i* with the per-package DOT value (as included in our data) and adding up these products. Following these patients over the 2011 to 2015 period, we observe a decreasing trend (Fig. 2C) and a decreasing correlation of *Z_i,t_* (Fig. 2D).

### Analysis

**Fig. 3.**
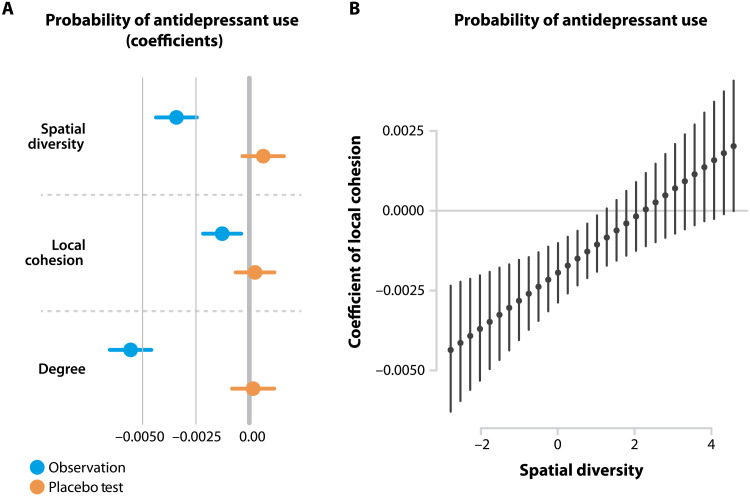
Local cohesion and spatial diversity of social networks can predict antidepressant use. (**A**) Spatial network structure predicts the probability of antidepressant use compared to randomized outcome (placebo). Coefficients of variables standardized to ζ-scores and 95% confidence intervals are plotted. (**B**) Local cohesion loses significance as spatial diversity increases. Coefficients of variables standardized to ζ-scores and 95% confidence intervals are plotted.

We first estimate the likelihood of antidepressant use with a linear probability model, in which *A_i_* is predicted with an ordinary least squares (OLS) regression including the main explanatory variables and further controls

$$P\left(A_{i}=1\right)=\alpha +\beta_{1}{\text{LC}}_{i}+\beta_{2}{\text{SD}}_{i}+\beta_{3}\text{ln}\left(d_{i}\right)+\beta_{4}F_{i}^{r}+\beta X_{i}+\beta S_{h}+D_{c}$$ (3)

where ln(*d_i_*) is the natural logarithm of ego-network degree (Degree in Fig. 3), which has been found to be associated with mental health (*6*), and *F_i_^r^* is the concentration of connections within a radius (here we apply *r* = 50 km) that has been used to measure isolation from distant opportunities (*34*). Age and Gender are important individual characteristics that correlate with mental health (*6*) and are denoted here by *X_i_*. A collection of settlement-level variables, including income or unemployment that we cannot control for on the individual level due to lack of data, are denoted by *S_h_*. *D_c_* denotes county dummies that aims to capture the spatial correlation of *A_i_*. The linear probability model allows us to analyze the interplay between LC*_i_* and SD*_i_* in explaining *A_i_*. We use standardized values of all variables.

We confirm that female gender (β = 0.020, *P* < 2 × 10^−16^) and age (β = 0.020, *P* < 2 × 10^−16^) are significant predictors of mental health in our sample, in line with previous findings (*6*). The economic status of the environment also plays a role as antidepressant use is less likely in towns where average income is relatively high (β = −0.001, *P* = 0.031) and tends to be significantly higher in towns where unemployment rate is high (β = 0.001, *P* = 0.026). We find that individuals with more reported friends are less likely to take antidepressants (*P* < 2 × 10^−16^) (Fig. 3A). Having a geographically bounded social network, unlike in the case of economic development (*34*), does not correlate significantly with mental health problems (*P* = 0.147). The regression table of the estimation is reported in section S6 where we also introduce and discuss a logistic regression specification; the logistic regression model explains the variation of *A_i_* with area under the curve (AUC) = 0.72 (see section S7).

We find that SD*_i_* has a stronger negative association (*P* = 2.4 × 10^−12^) with *A_i_* than LC*_i_* does (*P* = 0.005) but weaker than the relationship with the number of social connections (*P* < 2 × 10^−16^) (Fig. 3A). This finding that the relationship between mental health and bridging social capital is stronger than with bonding social capital, which has been previously thought to dominate the maintenance of mental balance, is robust across various forms of *C_i_* normalization, using a narrower set of antidepressants, narrowing down the sample to individuals who have at least 10 friends, and using a logistic regression framework. The relationship is significant considering more conservative thresholds (*P* < 0.01) that are necessary to compare our results using a magnitude larger sample to the previous survey literature. The results do not change if we control for the antidepressant use of social contacts (*P* = 2.82 × 10^−12^) (*6*) or for the distance to psychiatric centers (*P* = 0.422) (*42*). (Robustness is reported in section S8.) The placebo test is run on the identical sample in which the variable *A_i_* is randomly reshuffled. None of the coefficients in the placebo test are significant at the 5% level.

The negative relationship between *A_i_* and LC*_i_* is qualitatively different from the reversed U-shape association between *A_i_* and the aspatial clustering coefficient (reported in section S4), while the clustering among *i*’s friends outside of hometown that we call EC*_i_* (external cohesion) has no significant relationship with *A_i_* (*P =* 0.252). These findings underline the role of local bonding. Diversity, however, might be present in vicinity networks as well, depending on the size of adjacent population. Testing SD*_i_*^<20km^, a modified diversity measure that only considers links to other towns within a 20-km radius, we find a significant relationship (*P <* 0.000). Yet, unlike SD*_i_* that has a stable negative coefficient across all town populations in our sample, SD*_i_*^<20km^ loses significance in the largest towns. In section S9, we provide further details on how the role of network cohesion and diversity depends on town population and come back to this point in Discussion as well.

In the next step, we investigate the interplay between LC_*i*_ and SD_*i*_ to understand whether spatial bonding and spatial bridging complement each other for mental health as they do in the case of economic opportunities (*43*). We therefore estimate Eq. 3 with an additional interaction term between LC*_i_* and SD*_i_*. The positive coefficient of the interaction term is reported in section S10 and suggests that spatial dimensions of network cohesion and network diversity are not complementary in the case of mental health. Instead, SD*_i_* mitigates the relationship between LC*_i_* and antidepressant use.

We therefore estimate the marginal effect of LC*_i_* on *A_i_* at levels of SD*_i_*, a technique that provides detailed information about their interplay. We find that the negative relationship between *A_i_* and LC*_i_* is stronger at low levels of SD*_i_* than at medium levels and becomes insignificant at high levels of SD*_i_* (Fig. 3B). The finding is robust against alternative normalizations of *C_i_* (see section S10). This evidence suggests that the access to diverse groups in distant places reduces the importance of cohesive local groups for maintaining mental health.

Last, we analyze the dynamics of DOT (*Z_i,t_*) of those patients who took antidepressants in 2011 (*N* = 10,291). Because *Z_i,t_* is strongly correlated across subsequent years (reported in Fig. 2D), controlling for the value of *Z*_*i*,2011_ enables us to evaluate the relationship of LC and SD with the dose of antidepressants in subsequent years, conditional on current use of antidepressants. We estimate the following equation with OLS regression

$$\begin{array}{c} Z_{i,t}=\alpha +\beta_{1}Z_{i,2011}+\beta_{2}{\hat{A}}_{h}+\beta_{3}{\text{LC}}_{i}+\beta_{4}{\text{SD}}_{i}+\beta_{5}\text{ln}\left(d_{i}\right)\\ +\beta_{6}F_{i}^{r}+\beta X_{i}+\beta S_{h}+D_{c}+\epsilon_{i,t} \end{array}$$ (4)

where ${\hat{A}}_{h}$ is the predicted probability that the individual living in town *h* takes antidepressants as a function of her distance to the nearest psychiatric center, which has been argued to increase the access to treatment (*42*, *44*), and the ratio of antidepressant users in the region of the town that captures spatial inequalities of antidepressant use (as reported in Fig. 1B). The inclusion of ${\hat{A}}_{h}$ helps us mitigate the selection bias of unequal access to antidepressants. More details of ${\hat{A}}_{h}$ prediction can be found in section S11. The other covariates in Eq. 4 are identical to the ones in Eq. 3.

Table 1 presents regression results in a stepwise manner for *t* ∈ {2013, 2015}. Known predictors of mental health are introduced in Model (1) that confirms relatively faster recovery for male and relatively young patients. Adding spatial social network measures, we find that SD*_i_* has a significant negative correlation with *Z*_*i*,2013_, that LC*_i_* is not significant, and that the significance of ln(*d_i_*) (Degree in Table 1) is above the 5% level. This finding suggests that diverse connections to distant communities have a mitigating effect on antidepressant use. The findings do not change when we introduce ${\hat{A}}_{h}$ in Model (2). We then test the heterogeneous impact of SD*_i_* on vulnerable population in Model (3) by including its interaction with the Age and Male variables and find that younger individuals benefit more from spatial bridging, but there is no significant difference across gender in this respect. All relationships hold when *Z*_*i*,2015_ is used as dependent variable in Model (4). The coefficient of SD*_i_*^<20km^ is only significant at the 5% level but gains additional power in low-income towns in Model (5). Section S12 contains results for *t* ∈ {2012, 2013, 2014, 2015} and for an alternative dependent variable Δ*Z_i,t_* that captures the change in DOT between year 2011 and *t*.

**Table 1.** Days of therapy for patients who took antidepressants in 2011, OLS regression. Not reported variables are unemployment and friends within 50 km. Standard errors are in parentheses. Asterisks ***, **, and * denote significance at the 1%, 5%, and 10% level, respectively.

	(1)	(2)	(3)	(4)	(5)
	*Z*_*i*,2013_	*Z*_*i*,2013_	*Z*_*i*,2013_	*Z*_*i*,2015_	*Z*_*i*,2015_ (SD*_i_* < 20 km)
*Z*_*i*,2011_	1.393***	1.393***	1.393***	1.221***	1.231***
	(0.020)	(0.020)	(0.020)	(0.022)	(0.022)
Male*_i_*	−0.247***	−0.248***	−0.248***	−0.305***	−0.309***
	(0.050)	(0.050)	(0.050)	(0.053)	(0.054)
Age*_i_*	0.334***	0.333***	0.334***	0.312***	0.321***
	(0.027)	(0.027)	(0.027)	(0.029)	(0.030)
SD*_i_*	−0.071**	−0.068**	−0.122***	−0.138***	−0.078*
	(0.031)	(0.031)	(0.041)	(0.044)	(0.046)
LC*_i_*	−0.023	−0.022	−0.026	−0.021	−0.268
	(0.027)	(0.027)	(0.027)	(0.029)	(0.553)
Degree*_i_*	−0.057*	−0.055*	−0.054*	−0.039	−0.027
	(0.030)	(0.030)	(0.030)	(0.032)	(0.032)
Income*_h_*		0.021	0.019	−0.011	−0.023
		(0.043)	(0.043)	(0.045)	(0.046)
${\hat{A}}_{h}$		9.021*	9.315*	15.087***	15.840***
		(5.165)	(5.165)	(5.505)	(5.589)
SD*_i_* × Male*_i_*			0.010	0.085	0.068
			(0.053)	(0.056)	(0.061)
SD*_i_* × Age*_i_*			0.072***	0.066**	0.009
			(0.028)	(0.030)	(0.033)
*SD_i_* × Income*_h_*			−0.048*	−0.036	−0.062**
			(0.026)	(0.028)	(0.031)
Constant	−4.064***	−4.409***	−4.409***	−3.816***	−3.585***
	(0.176)	(0.265)	(0.265)	(0.282)	(0.637)
*N*	9,769	9,769	9,769	9,769	9,520
*R* ^2^	0.355	0.355	0.355	0.277	0.275
Adjusted *R*^2^	0.353	0.353	0.353	0.274	0.273

## DISCUSSION

In sum, we use nationwide datasets regarding antidepressant prescriptions and OSNs to analyze the relationship between social network structure and mental health care. Our data do not allow us to infer a causal relation. Distress, depressive symptoms, and anxiety are known to hinder social relations, even in online platforms where unobserved individual characteristics can affect the network size and structure. Our research does not substitute experiments that can reveal causal mechanisms needed for practical applications. Yet, the large scale of the data signals a previously undocumented correlation between the spatial diversity of social connections and mental health. This evidence suggests that bridging social capital at distant communities is important for mental health.

The theoretical importance of such bridging social capital has been previously highlighted by scholars who argue that engagement in more communities creates opportunity to develop connections of various functions, reduces isolation, and, consequently, helps maintain mental balance (*13*, *14*). Bridging social capital is extremely important for members of geographically isolated communities, where the lack of outside connections can make bonding social capital and cohesive social networks possibly even harmful for mental health by placing too much control on the individual (*5*, *45*) or by isolating the individual in an unhealthy social environment (*46*). Unlike the case of economic advantage, where diversity in social networks is thought to complement network cohesion (*43*), we demonstrate that diverse spatial networks across distant groups substitute local social bonding and might help patients with mental disorders to reduce the intensity of their treatment. These results contribute to recent calls for future research in geographical networks and health (*47*).

We also find that diverse connections to geographically distant communities might be a greater help for younger than for older individuals. This result might signal different usage of OSNs across generations such that younger cohorts can absorb and thus benefit more from diverse information than older cohorts (*48*, *49*). Yet, our data do not allow us to rule out alternative explanations. For example, younger individuals might be more active in establishing bridges across distant communities than older individuals, a mechanism that would require the analysis of dynamic social networks that we do not have. Individual-level information on income, unemployment, and further socioeconomic dimensions would also help us better understand how bridging ties can help disadvantaged and prospective groups and to avoid potential ecological fallacy (*50*). Such future research is important to inform health policy about social networking tools to help patients with mental disorders. Measuring tie strength is also an area for future work. Social media can also spoil mental health (*36*); thus, we need a better understanding how the role of online and offline communication networks changes over time. Whether our findings hold in large cities where local networks can provide more diversity is a question to be answered, ideally investigating fine mechanisms of social capital formation (*17*). Social capital and mental health are very likely related in population-scale networks.

## MATERIALS AND METHODS

### Data sources

The anonymized administrative dataset on antidepressant purchases has been provided by the Hungarian National Healthcare Service Centre. The data cover individuals who had at least one purchase of antidepressants or antibiotics over the 2011 to 2015 period. We use data on the purchase of medications in the ATC (Anatomical Therapeutic Chemical) group N06A (antidepressants) that were purchased through pharmacies; thus, hospital care is excluded. The exact type and amount of the medication purchased are known. We generate annual indicators of antidepressant use and fill in zero antidepressant prescription values for each individual for whom we do not observe antidepressant purchase in a given year. We obtain the gender, date of birth, and hometown from inpatient (hospital) and outpatient specialist care records of the patients included in the medication use data, covering years 2011 to 2015. The data consist of 9,017,323 individuals (of the total almost 10 million Hungarian citizens). The data are cleaned and curated by the Data Bank of the HUN-REN Centre for Economic and Regional Studies.

The OSN data cover information from individual profiles that were publicly available on the iWiW website. The platform was functioning from 2002 to 2014, and the entire dataset with basic user information (gender, date of birth, and hometown) and connection data (establishment of friendship ties) was accessed in 2012. By cleaning the data, we dropped those profiles that had no or more than 5000 connections and those that were not logged into after registration. Then, the investigated iWiW data include 2.7 million users who reported more than 300 million friendship ties by 2011. Further information about the data sources can be found in section S1.

### Methods

The data of antidepressant purchases were restricted to settlements with at most 20,000 inhabitants (4,728,045 individuals) but not less than 5000 inhabitants. Both the antidepressant use dataset and the social media dataset were restricted to those date of birth–town–gender cells, which have a single observation (2,163,158 individuals). Last, the restricted files have been matched by the date of birth–town–gender characteristics, resulting in 298,441 matched observations. Section S2 contains a discussion on the estimated probability of failure of the matching process.
