# Supplementary material for: Antidepressant use in spatial social networks
Source: Sci Adv. 2024 Dec 6;10(49):eadr0302. doi: 10.1126/sciadv.adr0302 (PMC11623294; doi:10.1126/sciadv.adr0302)
Supplement: Supplementary file 1 — Supplementary Text S1 to S13 Figs. S1 to S14 Tables S1 to S15 [file sciadv.adr0302_sm.pdf]

Science Advances
AAAS

Supplementary Materials for

Antidepressant use in spatial social networks

Balázs Lengyel *et al.*

Corresponding author: Balázs Lengyel, lengyel.balazs@krtk.hun-ren.hu

Sci. Adv. **10**, eadr0302 (2024)
DOI: 10.1126/sciadv.adr0302

This PDF file includes:

Supplementary Text S1 to S13
Figs. S1 to S14
Tables S1 to S15

S1. Data Sources

We use anonymized administrative data on antidepressant purchases for the entire population of Hungary, provided by the Hungarian National Healthcare Service Centre. The sample consists of individuals who had at least one purchase of antidepressants or antibiotics for systemic use over the 2011-2015 period. Based on an administrative data set on a random 50% of the Hungarian population (data available at the Data Bank of the Centre for Economic and Regional Studies), 89.7% of the population had at least one purchase of antidepressants or antibiotics for systemic use over 2011-2015.

The pharmaceutical records we use in this paper show data on the purchase of medications in the ATC (Anatomical Therapeutic Chemical) group N06A (antidepressants) that were purchased through pharmacies. The medication records relate only to the ambulatory setting and thus exclude hospital care. We know the exact type and amount of the medication purchased; hence days of therapy (DOT) can be calculated. We obtain the gender, date of birth, and settlement indicator from inpatient and outpatient care records covering years 2011-2015. These indicators are needed for matching the drug usage and our network (iWiW) data. Importantly, we then have the gender, date of birth, and settlement indicator for all individuals who purchased antidepressants or antibiotics for systemic use at least once and had an outpatient or inpatient care event in 2011-2015, resulting in a data set of 9,017,323 (Hungary had a population of 9,9 million in 2011). We generate annual indicators of antidepressant use and fill in zero antidepressant prescription values for each individual for whom we do not observe antidepressant purchase in a given year.

To capture spatial social networks, we use data from a Hungarian online social network called iWiW. This website was the market leader social media platform used by nearly 40% of the country's population. iWiW was launched in 2002 and shortly became the most widely used online social network in Hungary. At its peak around 2008-2010, it was one of the most visited national websites reaching the majority of internet users of the country. During the first few years of operation, iWiW provided only basic functionalities, mostly built around finding present and former friends, classmates, colleagues, and looking through one's acquaintance's' acquaintances. Later, photo upload, newsfeed, messaging, applet to visualize connections and the ability to develop external applications were introduced to the service. However, iWiW failed to follow leading social media portals in terms of hosting the increasingly intense online communication. Instead, iWiW remained a phone-book type online collection of friends.

Due to the increasing maintenance costs, low profitability and tough competition from Facebook, the site was closed down on June 30, 2014. Although the number of daily visitors begun to fall back significantly from 2011-2012, users rarely deleted their profiles: they just abandoned the service. In February 2013, the entire dataset of iWiW with basic user information (i.e., date of registration, gender, age, etc.) and connection data (establishment of friendship ties) were made

available for us for scientific research purposes. The self-reported location of users is available at the town level. We can analyze more than 300 million friendship ties the users have established by the end of 2011. Previous research demonstrated that geographical factors explain network structure (26) and found relationship between structural measures of social capital and outcomes such as the prevalence of corruption in towns (32) and dynamics of income inequalities in towns (33).

S2. Matching Social Media with Antidepressant Data

We restrict the data of antidepressant purchases to settlements with at most 20,000 inhabitants (4,728,045 individuals), to reduce the probability of false matches between the drug use and iWiW data. Next, we exclude individuals who live in towns where population size is less than 5,000 inhabitants because the fraction of iWiW users fell sharply in these smallest towns (33). We restrict both the drug user file and the iWiW data file to those date of birth – settlement – gender cells which have a single observation (2,163,158 individuals). We then match the so restricted drug user file to the iWiW file, which is now a 1:1 matching, resulting in 298,441 matched observations.

On the side of prescription data, the estimated probability of failure in the above matching procedure is low. If the drug user file covered the entire population, then the restriction to those date of birth – settlement – gender cells which have a single observation would ensure that there are no false matches when matching the data with the iWiW file. However, we do not observe approximately 9% of the population (for whom we do not have any medical records in 2011-2015). In a settlement of 20,000 inhabitants (the maximum in our restricted sample), we therefore do not observe on average 1,800 individuals. Assuming, for simplicity, that there are $65 \times 365 \times 2 = 47,450$ unique date of birth – gender combinations which are evenly distributed in the population, then there is a 3.7% probability that there is an individual among the 1,800 unobserved individuals who has the same date of birth – gender combination as a specific individual in the observed pool ($0.037 = 1 - (47,449/47,450)^{1,800}$). This is the probability of a false match that comes from the incomplete prescription data in the largest settlements in our sample, which is lower in smaller settlements. In fig. S10, we illustrate these false matching probabilities as function of town population, when we discuss how our findings might be different in large towns and cities.

Importantly, the mean annual days of therapy of antidepressants in 2011 does not differ substantially across the samples – in the entire drug use file it is 10.66, in the sample restricted to settlements with at most 20,000 inhabitants it is 10.58, and in the sample restricted to cells with single birth – settlement – gender observations it is 10.47.

On the side of the iWiW data, self-reported hometown and birthday information are the potential sources of noise. The hometown of users had to be chosen from a drop-down list when registering a profile on iWiW. This geography has already been repeatedly used in previous research on iWiW including the spatial structure of the network (26), and town-level correlations with indicators of corruption (32) and income inequalities (33). However, it might be possible that individuals register a profile in a town and forget to change their hometown when changing their official address after moving to another town. Further, it is also possible that people choose randomly from the drop-down list of towns. We check this latter potential source of noise by calculating the ratio

of observed user rate in the town (the number of registered iWiW users divided by total population) and the expected user rate (defined by the user rate divided by the ratio the town's population in the country population) (fig. S1.A). We find a relatively flat distribution of this indicator fluctuating around 1 across most letters that towns' names start with. Yet, the observed user rate is higher than the expected user rate in towns the name of which starts with 'Z'. This can be a potential source of noise; however, the majority of these towns are very small, which increases the observed ratio if only a few reports the town as home even though it is different officially. There are only two towns (Zalaszentgrót, Zirc) included in our matched sample out of all 68 towns that have a name with 'Z'.

Users had to report their age but were not forced to fill out their birthday data. In our specific sample, 512,050 users provided all year, month, and day of birth information out of all 765,956 users who live in investigated towns. The birthyear distribution reveals that users could register a profile from age 14 (fig. S1.B). The histograms of reported months do not show bias (fig. S1.C); however, the days of birth concentrate unexpectedly on the first days of the months (fig. S1.D), which might be due to reporting error. Therefore, we run the final regressions by dropping individuals who reported the first day of the month as birthday and following the same logic drop January births too. Results do not change significantly; we document these regressions in section S13.

S3. Town-level Analysis

Individuals in our sample reside in 409 towns. To demonstrate that this sample is not different from other towns in the country, we compare the distribution of the fraction of antidepressant users in sample towns and all other towns where we do not match the two data sources (N = 2219). The two-sided Mann-Whitney U test is not significant (p = 0.971) indicating that the sample towns where we performed the matching between the iWiW, and antidepressant data (N = 409) are not significantly different from the rest of the country. Besides correlation with antidepressant use, town-level socio-economic characteristics might introduce a bias into the measurement of social networks with the iWiW data. However, the fraction of iWiW users are significantly higher in sample towns compared to those that we don't analyze (fig. S2). Thus, iWiW better represents social networks in sample towns than elsewhere. Further, income per capita is higher, while unemployment rate and the fraction of citizens with elementary education as highest degree are significantly smaller in analyzed towns than in those that are not selected into the sample. These differences enable us to assume that citizens in sample towns turn to doctors in case of depressive symptoms with a higher likelihood than in other towns not analyzed.

Next, we investigate whether town characteristics are correlated with the fraction of antidepressant users. We find no correlation between population and fraction of antidepressant users (both variables are log-transformed) in the total set of towns ($r = -0.031$) (fig. S3). Ordinary least squares regressions confirm that population becomes significantly associated with the fraction of antidepressant users only when further socio-economic characteristics of the sample towns, such as average income, unemployment rate, and distance to the closest country border are controlled for (table S1). We observe that antidepressants are taken by slightly more individuals in relatively larger towns. More importantly, antidepressant use is significantly lower in relatively wealthier towns. Therefore, we control for the socio-economic characteristics of towns in the individual-level regressions that we present in Fig. 3 in the main text and in robustness checks in section S7.

S4. Aspatial Measure of Network Closure and its Relationship with Antidepressant Use

The Clustering Coefficient (C_i, defined as local clustering) measures the number of closed triangles in the network of the ego i, compared to all possible triangles in the ego-network. This indicator characterizes the node by capturing both network cohesion and diversity in a single scale. Low values of the indicator refer to diverse structure, while high values denote cohesive networks.

We test the correlation between C_i and antidepressant probability, estimating a linear probability model

$$P(A_i = 1) = \alpha + \beta_1 C_i + \beta_2 C_i^2 + \beta_3 \ln(d_i) + \beta X_i \quad \text{(S1)}$$

where $\ln(d_i)$ is the natural logarithm of ego network degree, and X_i stands for age and gender that are known determinants of antidepressant use (6).

We find an unexpected positive relationship between C_i and antidepressant use that signals a previously unknown importance of network diversity for mental health (table S2). However, the significant and negative coefficient of the squared term of C_i suggests a non-linear relationship.

Indeed, plotting the predicted antidepressant probability at values of the Clustering Coefficient by fixing all other variables at their mean, we find a reversed U-shape curve (fig. S4). This suggests that individuals with very diverse networks (very few of their friends know each other) or with very cohesive networks (most of their friends know each other) are less likely to take antidepressants than those individuals whose friendship networks contain 50% of all possible triangles.

The reversed U-shape relationship between network closure and antidepressant use has not been reported previously. However, this single-scale structural approach does now allow to disentangle the role of cohesive and diverse networks because it is difficult to say in a static online social network without node labeling whether network cohesion and diversity is measured within or across socio-economic or demographic groups. In this article, we use the spatial dimension of social connections to investigate how cohesive networks within towns and diverse connections across towns correlate with mental health that help us disentangle the role of bonding and bridging social capital.

S5. Variables and Descriptive Statistics

Research has consistently shown that close social relationships are beneficial for maintaining good mental health. Being surrounded by a community that one can rely on provides a sense of security and confidence, which can assist in managing anxiety. In the social capital literature, this idea is often operationalized through clustering, also known as transitivity concerning social relationships. Clustering refers to the proportion of closed triples in a social network. Higher transitivity values foster trust and facilitate the creation of cohesive communities. In our research, we extend the conventional clustering calculation by incorporating spatial considerations and, more specifically, examining the degree of social cohesion within one's immediate living environment. This concept is referred to as Local Cohesion and is denoted by LC_i.

Another crucial variable that is rather new in the literature of empirical research on mental health is social network diversity, particularly in terms of its spatial dimension. Thus far, research has not been able to explore individuals' weak connections and their potential correlations with mental health outcomes. While some studies have shown that a more diverse communication telephone network is associated with higher financial well-being (16), the effects of social network diversity on mental health have not been extensively examined. In our study, we incorporate Spatial Diversity as a variable denoted by SDi into our regression analysis.

To compute these indices, we first measure the local clustering coefficient of individuals in their friendship networks within their hometown that is the basis of the Local Cohesion variable and the entropy of friendship ties across towns that is the basis of the Spatial Diversity variable. Both measures follow a distribution that is close to normal (fig. S5). Yet, these measures can be correlated with degree and can be influenced by the number connections within and across towns, the triadic closure outside of hometown and the number of towns the individual is connected to. Therefore, we apply two normalization methods that enable the comparison of the network structure among individuals with very different number of friends in their hometown and in other towns as well.

Local Cohesion is quantified by dividing the local clustering of individuals within their home-town network by the local clustering of a series of randomly rewired networks that keep the degree of ego and the number of links in the ego network (fig. S6). This procedure randomizes links in the network of individuals without considering town borders. Therefore, Local Cohesion captures the intensity of triadic closure within hometown compared to random alternative scenarios in which the probability of friendship is identical, but triangles are randomly closed within and across towns. Thus, our Local Cohesion measure is bigger than 1 if the closed triangles are concentrating within the town, compared to random triangles.

Next, Spatial Diversity is calculated by dividing the entropy of social connections across towns by the number of towns that the individual has access to (fig. S7). This procedure is the standard method to compare diversity of high degree and low degree individuals (16).

The above normalization procedure does not induce a radical change to the entropy distribution; Spatial Diversity is close to normal. However, the distribution of Local Cohesion becomes right-skewed indicating that local clustering in hometown are similar to randomly rewired networks for most individuals in the sample (fig. S8).

The individual-level analysis contains further network variables and demographic controllers. Degree measures the log-transformed number of friends on iWiW that has been repeatedly shown to correlate with mental health (36). Social networks in remote areas in Hungary are frequently concentrated within close geographical proximity (26) that can hinder access to diverse information (33). Thus, we include the fraction of friends within a specified radius, denoted by r. In the estimation model, r has been set to 50km that reflects meaningful spatial scales in Hungary. To capture the relationship between the number of friends and antidepressant use, we take the logarithm of the former variable.

Age and gender are also included as important determinants of mental health. fig. S8 depicts the distribution of these variables and table S3 presents their descriptive statistics. Mental disorders have been shown to propagate in social networks through peer effects (6). Therefore, we create a binary variable "Friend taking Antidepressant" that takes the value of 1 if the individual has at least one friend who takes antidepressants and is 0 otherwise. The median value of this indicator is 1, which means that most of the individuals in our sample know at least one person who takes antidepressants (table S3). Unlike in previous studies, we now investigate a network where weak ties are abundant, probably channeling different mechanisms of mental disorder peer effects than strong ties. Therefore, we use this variable only in robustness checks.

Pearson correlation values of independent variables are low in most cases (table S4). Degree is negatively correlated with Local Cohesion implying that individuals who have many friends tend to have local clustering in their hometown that is relatively similar to random networks. There is a positive correlation between Degree and Friend taking Antidepressant because the more friend one has the higher likelihood that one of them will use antidepressants. None of these correlations are strong enough to impose problems (variance inflation) of severe multicollinearity.

S6. The Probability of Antidepressant Use: Estimation Results

To examine the relationship between mental health and spatial social networks, we utilize multivariate regression analysis. Our binary dependent variable, A_i is equal to 1 if the individual purchased a prescribed antidepressant in 2011, serving as a useful proxy for mental health problems. Although the exact form of the latent variable is unknown, regressions using drug use as a proxy provide an adequate approximation of the probability of depression. The linear probability model (LPM) has increasing popularity in social sciences because it allows for a straightforward interpretation of the interaction term between explanatory variables that is important for our line of argument. However, using the LPM requires the assumption of linear relationship between social network structure and the probability of antidepressant use that we cannot test. Thus, we also employ a logistic regression estimation framework as a robustness check.

Besides the LPM regression that is specified in Equation 3 in the main text, we also estimate a logistic regression model as follows:

$$ln\left(\frac{P(A_i=1)}{1-P(A_i=1)}\right) = \alpha + \beta_1 LC_i + \beta_2 SD_i + \beta_3 \ln(d_i) + \beta_4 F_i^r + \beta \mathbf{X}_i + \beta \mathbf{S}_h + \mathbf{D}_c \qquad \text{(S2)}$$

where $P(A_i = 1)$ captures the probability that individual is an anti-depressant user, $ln(d_i)$ is the natural logarithm of degree, and F_i^r is the fraction of friends within a radius $r = 50km$. Individual-level demographic variables Age and Gender are denoted by $\mathbf{X}_i$, a collection of settlement-level variables is denoted by $\mathbf{S}_h$, and $\mathbf{D}_c$ denotes county dummies. We include age and gender as control variables as these are expected to be related to the risk of depression, and to the network characteristics. Moreover, we recognize that many individual-level characteristics contribute to mental balance, including income or unemployment. However, we do not have individual-level data on income and unemployment; therefore, we control for them on the level of towns.

table S5 presents the estimation results. Besides the LPM findings reported in the main text, one can observe that the concentration of friendship ties has no significant relationship with antidepressant use. As expected, men are significantly less likely to take antidepressants than women and the likelihood increases as the age grows; these findings are in line with previous results (7). Further, we find that individual antidepressant use is less likely in towns where average income is relatively high and more likely where the unemployment rate is relatively high.

The Placebo test is run on an identical sample in which the variable A_i is randomly reshuffled. None of the coefficients are significant, verifying that the results are not an artefact of chance.

Finally, we find that the logistic regression confirms the robustness of almost all variables. LC_i is an exception as it's negative relationship with A_i is not significant in the logistic regression. Yet, the relationships of SD_i and degree remain significant.

S7. Predictive Accuracy

The quality of LPM fit is usually very low. Therefore, we measure estimation precision of the logistic regression and focus on determining the confidence level at which we can differentiate between antidepressant users and non-users, referred to as "model accuracy", using our model specified in Equation S2.

To assess the model accuracy, we calculate two key metrics: Sensitivity and Specificity. Sensitivity, or the true positive rate can be calculated from true positives (TP) and false negative (FN) by the formula $TP/(TP + FN)$. This indicator measures the proportion of individuals correctly classified as antidepressant user by the logit regression model. It reflects the probability of obtaining a positive test result when the individual is genuinely a user. Specificity, the true negative ratio, is calculated from true negatives (TN) and false positive (FP) by $TN/(FP + TN)$ and measures the proportion of individuals accurately classified as non-user. It signifies the probability of obtaining a negative test result when the individual is genuinely not using antidepressants.

To visualize the model's ability to distinguish between antidepressant users and non-users, we construct a receiver operating characteristic (ROC) curve (fig. S9). This graphical representation plots the accuracy ratios of Sensitivity and Specificity. The ROC curve's 45-degree reference line denotes the accuracy achieved by random chance in classifying binary observations. A greater deviation of the ROC curve from the reference line indicates a higher discriminatory capability of our estimator. The area under the ROC curve provides a numerical measure of the predictive accuracy (AUC). Our logit regression model achieves a predictive accuracy of 72% in distinguishing between antidepressant users and non-users.

Establishing a consensus on what constitutes a sufficiently high predictive accuracy value is challenging. Nonetheless, within the social sciences realm, our achievement of 72% accuracy using social network variables and basic demographic indicators is considered notable. This finding underscores the potential of these variables to accurately discern between antidepressant users and non-users, contributing to advancements in mental health research.

S8. Robustness

To ensure the robustness of our LMP and logistic regression models, we conducted several additional multivariate analyses. table S6 with Linear Probability and S7 with Logit models document these and contain the main model too (Model 1 in both tables). In Model 2 of both table S6 and table S7, the indicator of antidepressant use is restricted to those prescriptions on which the first digit of the ICD-10 (International Classification of Diseases) code is “F”, indicating mental, behavioral and neurodevelopmental disorders. This can be considered as a clean indicator of antidepressant use that is surely related to mental health problems. However, the reliability of diagnosis codes on the prescriptions are limited by the fact that diagnosis code on the prescription has no impact on the cost or any other aspects of the medication or on the following therapy. Moreover, a prescription can be used even if the diagnosis code is missing, which is indeed the case for 5% of the prescriptions in our raw data. In this specification, the negative association between Local Cohesion, Spatial Diversity and Antidepressant Use do not change compared to the main model.

Recognizing the potential bias associated with smaller network sizes, we restricted our sample to individuals who had at least 10 acquaintances in the online social network. The results of this restricted sample analysis are presented in Model 3 in both tables and provide insights into the impact of network size on our findings, we see that all variables remained unchanged.

Building upon the notion that depression can be influenced by social norms and the behavior of acquaintances, similar to previous research on the influence of friends’ obesity on individual mental health (6), we incorporated a control for the presence of depressed individuals within our social network. In Model 4, we observed a significant peer effect, indicating the influence of peers on an individual’s likelihood of taking antidepressants. Our main variables remain unchanged. However, since our study design lacks temporal information about the friendship ties, establishing causality remains a challenge. This presents an important avenue for future research to explore the dynamics of depression transmission.

In Model 5 of both tables, we examined the role of accessibility to psychiatric clinics, specifically focusing on the distance an individual has to travel to reach the nearest clinic with psychiatric services. Our expectation was that individuals living closer to clinics would have better access to diagnosis and antidepressant medication (42). Surprisingly, the results did not confirm this hypothesis, suggesting that proximity to a clinic did not significantly influence the likelihood of depression diagnosis in the presence of social network variables.

Additionally, we introduced a variable capturing the distance to the nearest border in Model 6 of both tables. Given Hungary’s unique historical context, where settlements with Hungarian citizens lie beyond the borders of neighboring countries and fall outside the coverage of the

Hungarian health insurance system, we anticipated potential limitations in observing social contacts and network diversity in these border areas. However, the results did not reveal any substantial border relationship with antidepressant use. Model 7 includes a town-level variable that captures the fraction of population with the elementary school as highest degree. As expected, this shows a negative correlation of antidepressant probability, because poorly educated people are less likely to see a doctor in case of depressive symptoms.

By conducting these robustness checks, we have further examined the stability and generalizability of our main results. Despite variations in sample restrictions, the inclusion of additional variables, and the choice of alternative regression methods, our findings consistently support the previously unknown relationship between Spatial Diversity as a measure of bridging social capital and Antidepressant Use, providing confidence in the reliability of our results. Local Cohesion has a consistent significant relationship with Antidepressant Use in the linear probability models but the variable loses significance in the logit models.

S9. Network Diversity and Cohesion in and across Towns

In this paper, we consider geography as a major driver of social group formation. Specifically, we argue that cohesive social connections within hometowns signal bonding social capital of the ego while diverse connections across towns suggest bridging social capital. Here we provide supporting information for the above argument.

First, we discuss the relationship of diversity in spatial social networks and town size. Undoubtedly, large towns provide more opportunities to develop diverse connections within the settlement than small towns. In this respect, our data matching exercise limits our abilities to understand how much inter-city connections are needed for mental health compared to intra-city bridging ties, especially in large towns and cities. The probability of false matching increases from 0.01% to 0.04% as population grows from 5K to 20K in our town sample and continues to rise quickly in larger towns (fig. S10). However, the fragmentation of the networks within towns – a measure that quantifies the extent to which social relations tend to concentrate in tightly-knit communities compared to bridging ties (33) – does not change substantially in the range of towns that exceed our upper population limit. This suggests that including large towns in our sample would risk high likelihood of false matching but individuals – on average – would not have different social networks within towns.

To test whether social connections within and across towns are appropriate predictors of antidepressant use, we repeat the regression specified by Equation 3 in the main text by applying alternative network measures. *External Cohesion* (EC_i) is the clustering coefficient of *i*'s ego-network outside her hometown, normalized by the Erdős-Rényi randomization procedure that we used in the case of LC_i as well. This alternative EC_i indicator has no significant relationship with antidepressant use (table S8) that provides support for our argument that local connections can be used to measure bonding social capital.

Next, we measure SD_i from connections to towns that are within 20km radius of the hometown of *i* ($SD_i^{<20km}$) or, on the contrary, are further away ($SD_i^{>20km}$). Since our spatial approach cannot be applied within towns, and measuring bridging ties across groups defined by other dimensions (like age or gender groups) would stretch the limits of this paper, we have chosen this spatial threshold that is often used to capture commuting zones in the Hungarian context. We find that $SD_i^{<20km}$ is negatively correlated with antidepressant use, while $SD_i^{>20km}$ is not. This result implies that bridging ties across proximate towns can already provide diversity that can be beneficial for mental health.

However, while the coefficient of SD_i is significant in the entire range of or town sample, the significance of $SD_i^{<20km}$ vanishes as town population grows (fig. S11).

S10. Interaction of Local Cohesion and Spatial Diversity

Interaction terms between variables are commonly used for estimating a variable's conditional effect on another's contribution to the outcome. However, interpreting an interaction term is not as simple as interpreting a coefficient. In the following model, we utilize an interaction term to estimate the conditional effect of a given level of Spatial Diversity SD_i on the contribution of Local Cohesion LC_i to the prediction of our dependent variable, the probability of antidepressant use:

$$P(A_i = 1) = \alpha + \beta_1 LC_i + \beta_2 SD_i + \beta_3 (LC_i \times SD_i) + \beta_4 \ln(d_i) + \beta_5 F_i^r + \beta \mathbf{X}_i + \beta \mathbf{S}_h + \mathbf{D}_c \qquad \text{(S3)}$$

where $LCi \times SDi$ is the interaction term.

table S9 presents regression results including interaction terms and contains three models that differ by the way how Local Cohesion variable is normalized. Model 1 contains the Local Cohesion variable that we discuss in the main text. In Model 2, we use an alternative measure that is the local clustering normalized by degree. Model 3 contains the non-normalized value of local clustering. The negative sign and significance of main explanatory variables are persistent across the models. However, the interaction term is significant only in Model 1 and Model 2. The positive value indicates that Spatial Diversity mitigates the relationship between Local Cohesion and Antidepressant Use.

However, we are not directly interested in the coefficients and the standard errors of the model parameters β_1, β_2 and β_3 etc., per se. In this case the contribution of LC_i to P_i could be estimated by the marginal effect:

$$\frac{\delta P_i}{\delta LC_i} = \beta_1 + \beta_3 SD_i, \qquad \text{(S4)}$$

while the standard error of marginal effect is estimated as:

$$\hat{\sigma}\left(\frac{\delta P_i}{\delta LC_i}\right) = \sqrt{var(\hat{\beta}_1) + SD_i'^2(\hat{\beta}_3) + 2SD_i cov(\hat{\beta}_1, \hat{\beta}_3)}. \qquad \text{(S5)}$$

Based on Equation S5, previous statistical studies have shown that we cannot rule out the possibility of a statistically significant contribution of LC_i on P_i for certain values of SD_i, even if all other model parameters are insignificant. This means that we cannot determine the real conditional effect of LC_i on P_i solely based on the effect sizes and standard errors of β_1 and β_3.

In order to address this issue, we follow the literature and calculate the marginal effect of LC_i at all possible values of SD_i.

The main text contains the marginal effect of Local Cohesion on Antidepressant Use at levels of Spatial Diversity. fig. S12 complements this with Local Cohesion indices normalized or not. The degree-normalized LC_i indicator behaves similarly as the Erdős-Rényi normalized variable. This result implies that the importance of Local Cohesion decreases and becomes insignificant as the value of Spatial Diversity increases. However, the non-normalized version of local clustering does not show this pattern.

S11. Access to Antidepressants as Selection Mechanism

The accessibility to healthcare might introduce a selection bias because those who have higher accessibility are more likely to purchase antidepressants; consequently, antidepressant use might be systematically under-measured in low accessibility communities. To control for this selection bias, we measure the distance to the nearest psychiatric center, that has been found to increase medical treatment intensity (42, 44). We argue that the ratio of antidepressant users in the region of the town is also a good measure to sort out the accessibility bias, for two reasons. First, only small-town residents are included in the analysis; thus, they do not have a dominant weight among antidepressant users in their larger regions. Second, the ratio of antidepressant users is spatially correlated such that high versus low ratio areas are far from each other, as it was reported in Fig. 1B.

To mitigate the selection bias of unequal access to antidepressants, we estimate the probability of antidepressant use A_i with the following logistic regression:

$$ln\left(\frac{P(A_i=1)}{1-P(A_i=1)}\right) = \beta_1 + \beta_2 Dist_h + \beta_3 A_r \quad \text{(S6)}$$

where $Dist_h$ is the Euclidean distance from home-town h of patient i to the closest psychiatric centre and A_r is the ratio of antidepressant users in the home-region r of patient i ($h \in r$). table S10 demonstrates that, unlike in previous research where distance to healthcare institutions were correlated negatively with treatment intensity, $Dist_h$ has a significant positive, but weak correlation with the probability of antidepressant use. The reason behind this finding is that psychiatric centers are located in regional centres and antidepressant use is typically higher in more remote towns that are further away from these central places. Next, we find that A_r is a significant and strong predictor of antidepressant use.

Next, we predict the probability of antidepressant use by using the point-estimates of $Dist_h$ and A_r. fig. S13 illustrates the distribution of the predicted value ($\hat{A}_h$). By including $\hat{A}_h$ in the probability of A_i estimations in the main text, we control for the potential bias of the spatial biases of antidepressant access. In table S11, we confirm that this inclusion does not eliminate the sign and significance of our predictors that we reported in Fig. 3 in the main text. It however does decrease the significance of the predictors when regressing the Days of Treatment. Therefore, we include $\hat{A}_h$ in the dynamics of dosage regressions that we report in Table 1 in the main text.

S12. Dynamics of Days of Therapy

We observe the quantity of purchased antidepressants that enables us to examine the impact of spatial social networks on the dynamics of antidepressant use by focusing on antidepressant users only. $Z_{i,t}$ is the Days of Therapy (DOT). To obtain the DOT indicator, for each antidepressant type we multiply the volume of antidepressant packages purchased in year t by individual i with the per-package DOT value (as included in our data), and add up these products. The distribution of the log-transformed $Z_{i,t}$ illustrates that most patients purchase a quantity that covers almost the entire year (the median is around $10^{2.5}$ = 315 days) (fig. S14). $Z_{i,t}$ is strongly correlated across subsequent years (reported in Fig. 2D in the main text). Thus, running regressions on the level of $Z_{i,t}$ by controlling for the value of $Z_{i,2011}$ enables us to evaluate the forms of spatial social networks in potentially mitigating the dose of antidepressants. We test the following equation with OLS regressions for $\{t \in 2012, 2013, 2014, 2015\}$:

$$Z_{i,t} = \alpha + \beta_1 Z_{i,2011} + \beta_2 LC_i + \beta_3 SD_i + \beta_4 \ln(d_i) + \beta_5 F_i^r + \beta \mathbf{X}_i + \beta \mathbf{S}_h + \mathbf{D}_c + \varepsilon_{i,t} \quad \text{(S7)}$$

where $Z_{i,2011}$ is the Days of Therapy in year 2011, the other co-variates are identical to the ones that we used in preceding analyses, and $\varepsilon_{i,t}$ is the error term.

As expected, $Z_{i,2011}$ is strongly correlated with $Z_{i,t}$ for every t (table S12 and S13). We also find the expected significant negative coefficient of the Male variable and the significant positive coefficient of the Age variable. Degree has a negative coefficient but is only significant at the 5% level only at t = 2014.

We find that Local Cohesion does not but Spatial Diversity does have a significant negative correlation with $Z_{i,t}$. This finding confirms that bridging social capital might have a mitigation effect on the mental disorders that is reflected by decreasing Days of Therapy. Next, we regress $\Delta Z_{i,t}$ that is the change of Days of Therapy between year 2011 and t, using the same variables:

$$\Delta Z_{i,t} = \alpha + \beta_1 Z_{i,2011} + \beta_2 LC_i + \beta_3 SD_i + \beta_4 \ln(d_i) + \beta_5 F_i^r + \beta \mathbf{X}_i + \beta \mathbf{S}_h + \mathbf{D}_c + \varepsilon_{i,t} \quad \text{(S7)}$$

These final results confirm previous findings (table S14). The correlation of $Z_{i,2011}$ drops as well as the R^2 of the models. Yet, the coefficients of Spatial Diversity, Degree, Male, and Age remain almost unchanged.

S13. Regression results by dropping potentially noisy observations

In section S2, we reported that the iWiW birthday data might include some noise, because the January months and first days of the months are overrepresented. Therefore, we re-run regressions without these observations to reproduce main findings in Fig. 3 and Table 1 in the main text. Results confirm previous findings (table S15).

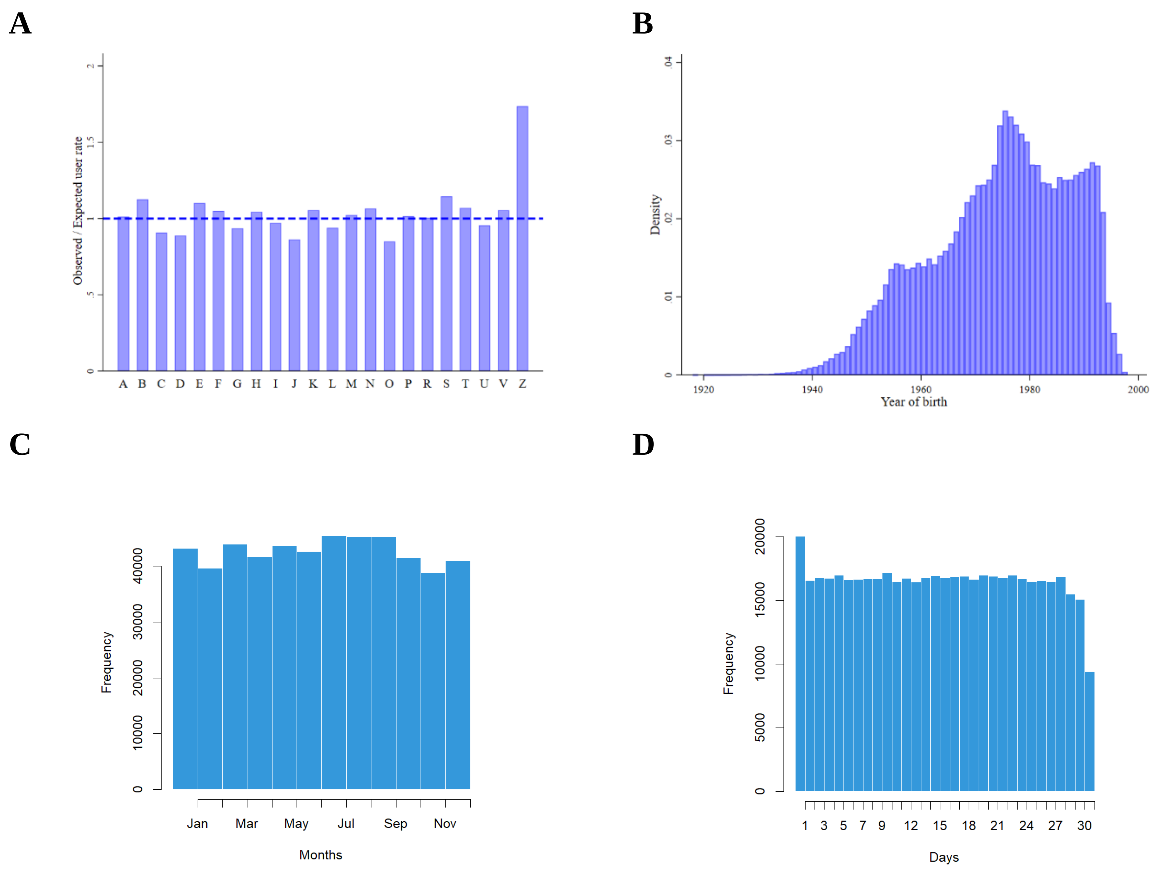

fig. S1.

Potential sources of matching noise from the iWiW data. **(A)** The ratio of observed and expected user rate by the first letter of the towns' name. **(B)** Birthyear distribution of iWiW users. **(C)** Reported months of birth on iWiW. **(D)** Reported days of birth on iWiW.

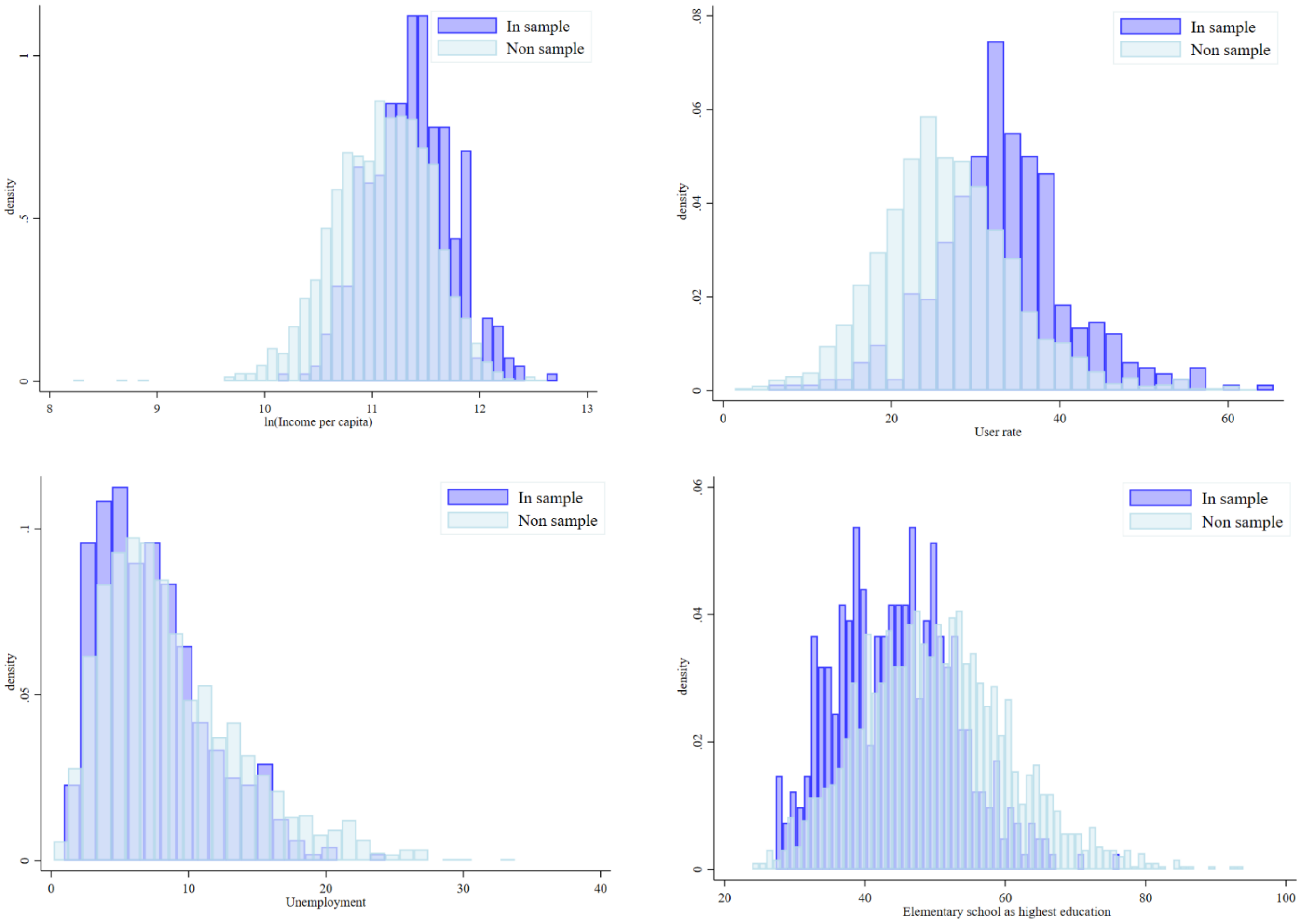

fig. S2.
Income, online social network user rate, unemployment and education by towns in sample.

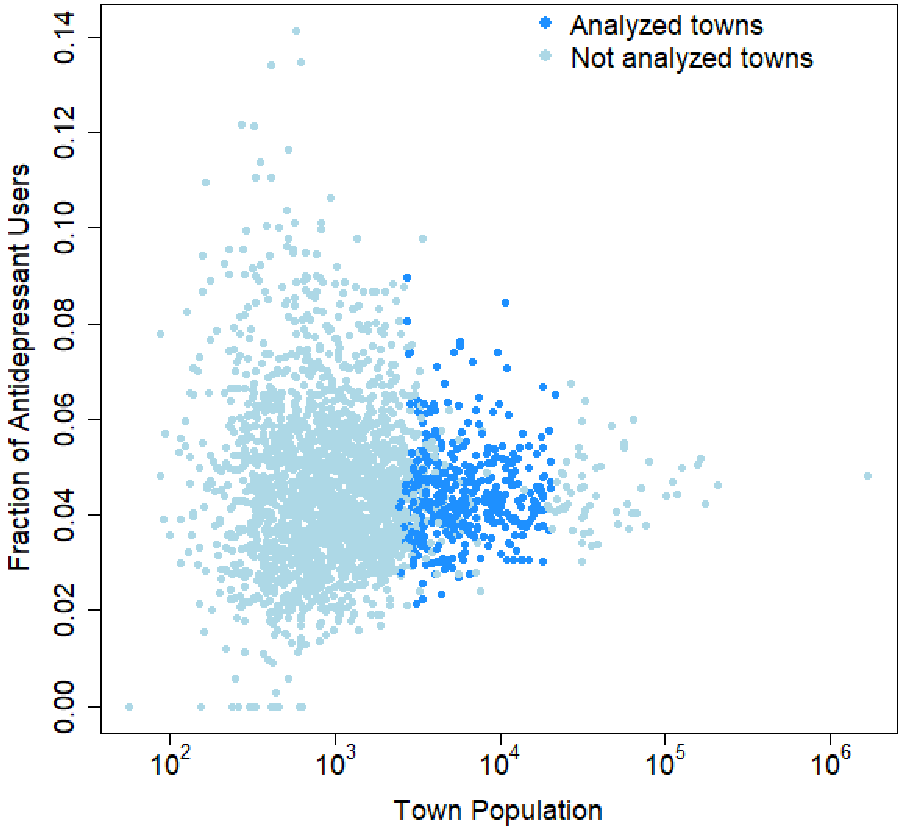

fig. S3.

Antidepressant usage is not correlated with town population.

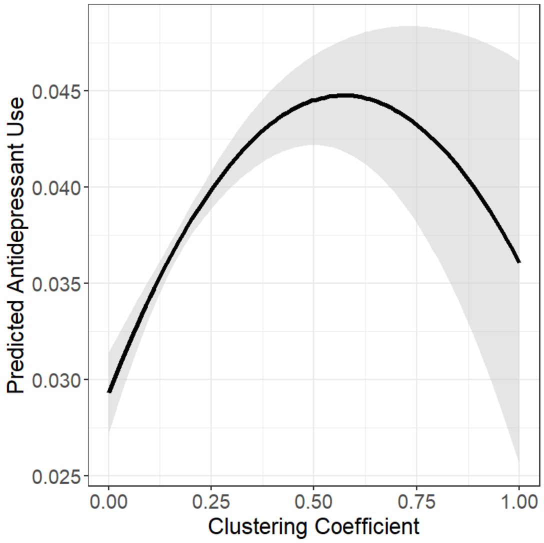

fig. S4.

The non-linear relationship between the clustering coefficient and antidepressant probability suggests a dual role of network cohesion and diversity.

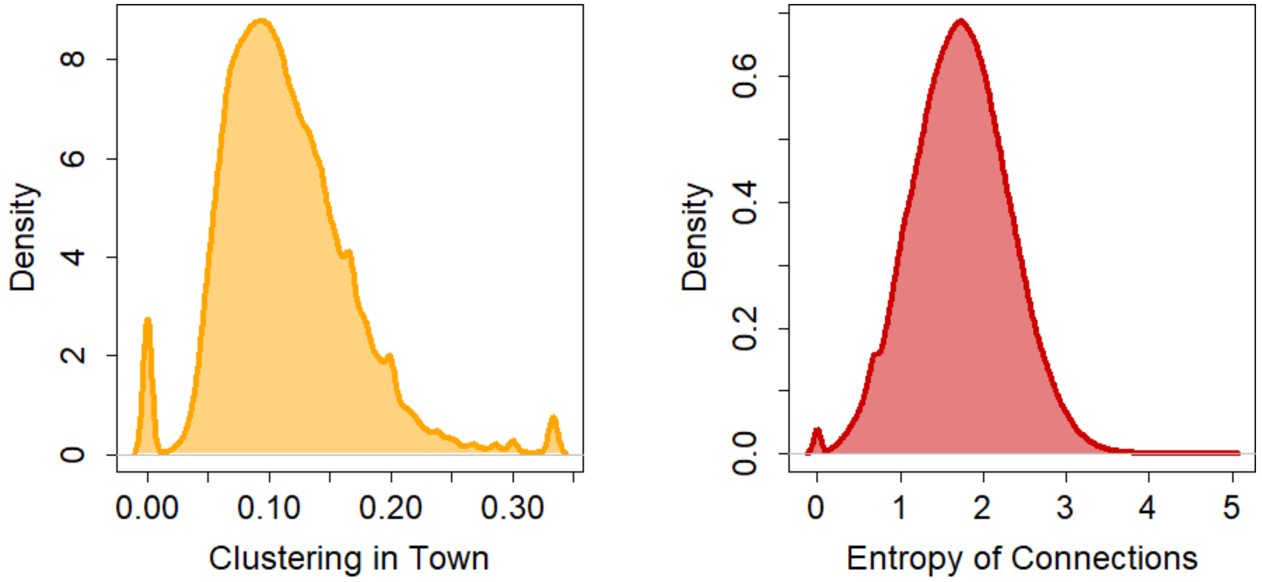

fig. S5.

Distribution of the non-normalized values of local clustering in the town of Ego and the entropy of Ego's connections across towns.

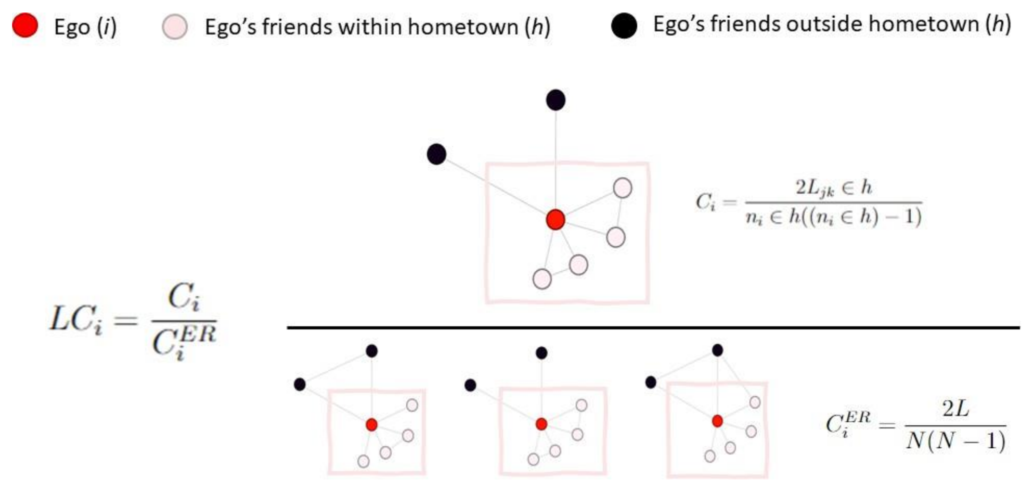

fig. S6.

Calculation of the Local Cohesion variable. The local clustering is compared to randomly rewired networks that keep degree and ego network density but do not consider town borders.

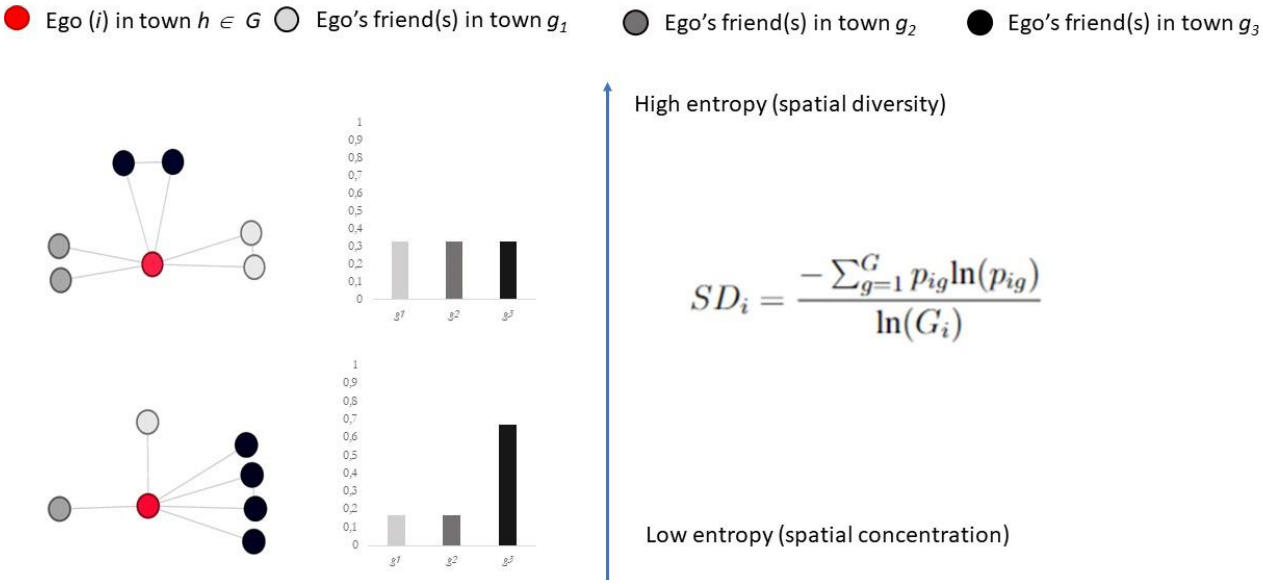

fig. S7.
Calculation of the Spatial Diversity variable. The entropy of connections across towns is divided by the number of towns the individual has access to.

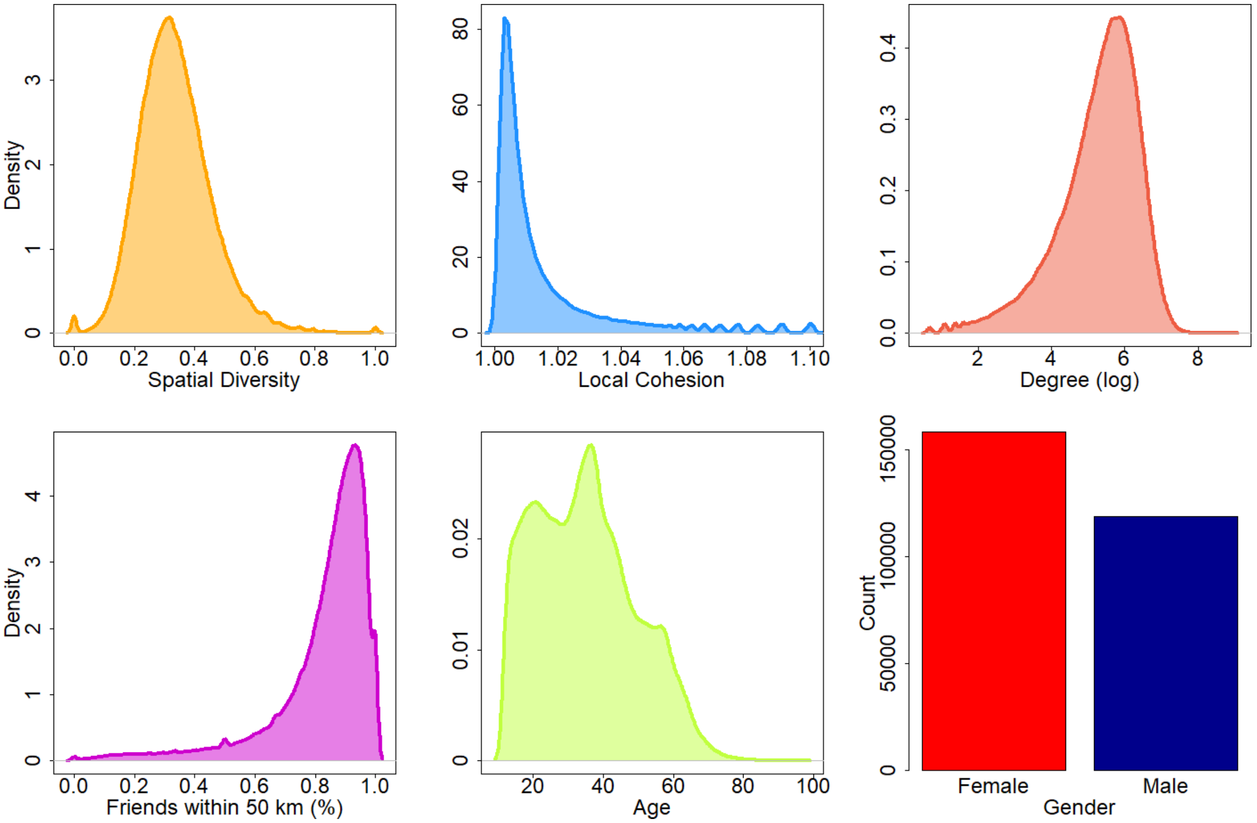

fig. S8.

Distributions of individual-level variables.

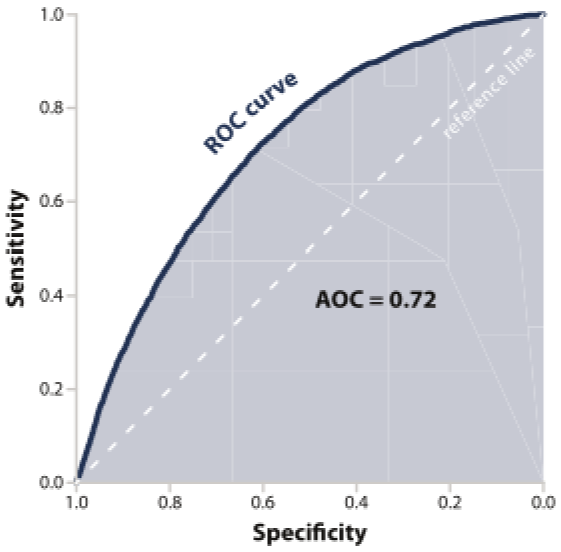

fig. S9.

Predictive accuracy of the logistic regression that estimates the Probability of Antidepressant Use.

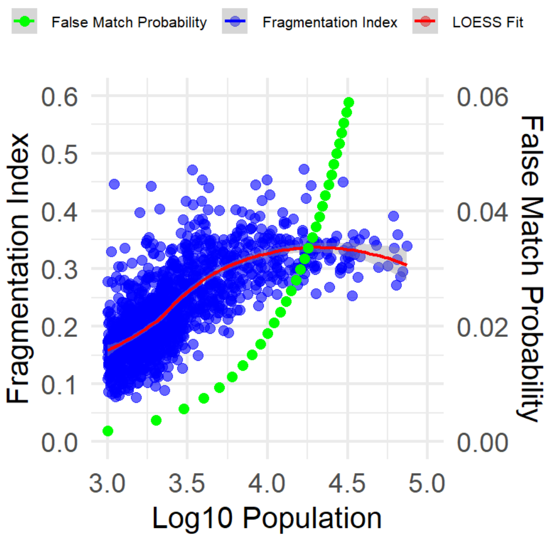

fig. S10.

Town size, social network structure and false matching probability.

A **B**

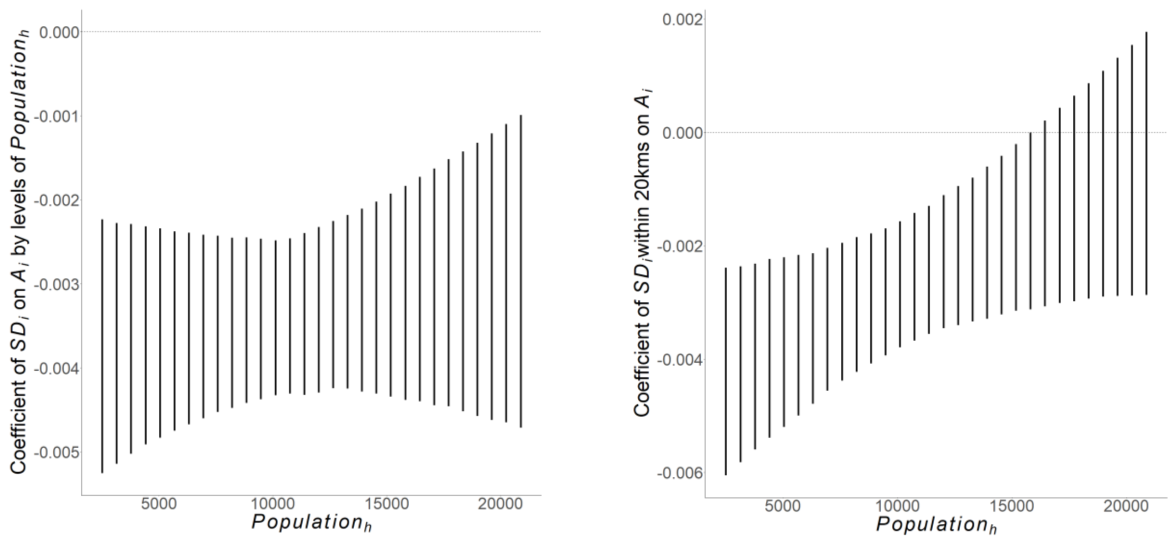

fig. S11.

The coefficient of SD_i on A_i by town population. **(A)** The coefficient is consistently negative and significant across town population ranges in our sample. **(B)** The coefficient of SD_i to towns that are within a 20km reach is significant negative in relatively small towns but it is not significant in the largest towns of our sample.

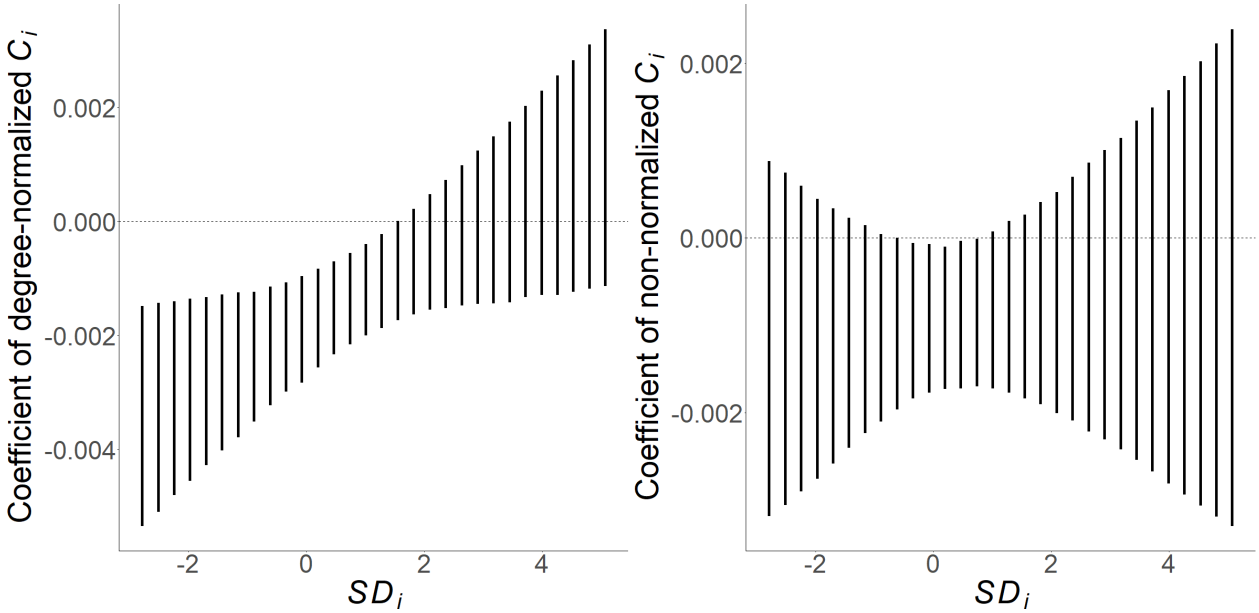

fig. S12.

Marginal effect of alternative measures of Local Cohesion on the Probability of Antidepressant Use by levels of Spatial Diversity.

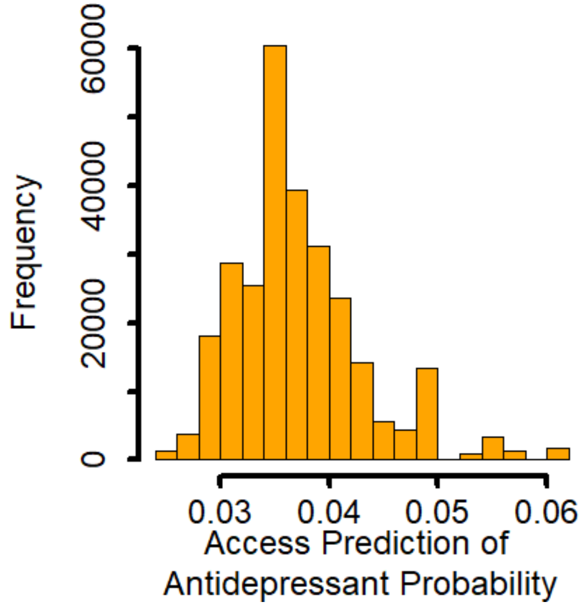

fig. S13.
Distribution of predicted values of antidepressant use by Distance to psychiatric centers and Antidepressant usage rate in the region of the individual.

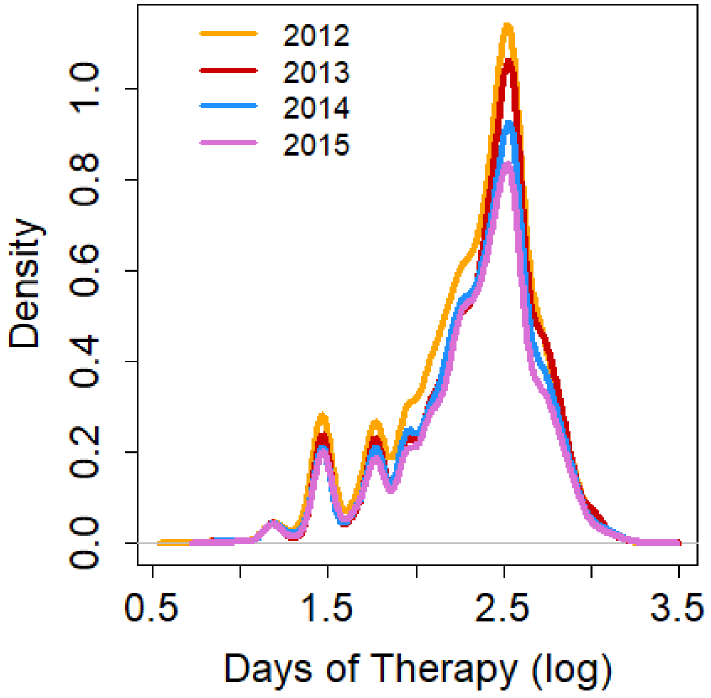

fig. S14.
Distribution of Days of Therapy reveals similar patterns over the analyzed period.

table S1.

Fraction of antidepressant users and town characteristics. Ordinary Least Squares regression.

	Fraction of Antidepressant Users (%, log)			
	M1	M2	M3	M4
Population (#, log)	-0.001	0.017	0.038**	0.038**
	(0.000)	(0.019)	(0.019)	(0.018)
Average Income (HUF, log)			−0.198***	−0.192***
			(0.036)	(0.036)
Unemployment Rate (%, log)				−0.071
				(0.350)
Distance to Border (km, log)				−0.029***
				(0.009)
Constant	0.048***	−3.274***	−0.807*	−0.772
	(0.001)	(0.163)	(0.479)	(0.478)
Observations	2624	409	409	409
R^2	0.001	0.002	0.070	0.092
Adjusted R^2	0.000	−0.001	0.065	0.083

Note: Standard errors in parentheses. *p<0.1; **p<0.05; ***p<0.01

table S2.

The probability of antidepressant use predicted by the Clustering Coefficient. Ordinary Least Square regression. Unreported controls include Age (numeric), and Male (dummy).

	Probability of Antidepressant Use	
	(1)	(2)
C_i	0.003^{***}	0.004^{***}
	(0.000)	(0.001)
$C_i{}^2$		-0.001^{***}
		(0.000)
$ln(d_i)$	-0.002^{***}	-0.002^{***}
	(0.000)	(0.000)
Constant	0.046^{**}	0.047^{**}
	(0.002)	(0.002)
Controls	Yes	Yes
Country FE	Yes	Yes
Observations	276,386	276,386
R^2	0.021	0.021

Note: Standard errors in parentheses. $^{*}p<0.1$; $^{**}p<0.05$; $^{***}p<0.01$

table S3.

Descriptive statistics of individual-level variables

Variables	N	Mean	SD	Median	Min	Max
Antidepressant Use	277,279	0.03	0.19	0.00	0.00	1.00
Spatial Diversity	277,279	0.34	0.12	0.32	0.00	1.00
Local Cohesion	265,719	1.03	0.06	1.01	1.00	1.50
Degree (log)	277,279	5.27	1.10	5.48	0.69	8.87
Friends within 50 km	277,344	0.83	0.17	0.87	0.00	1.00
Male	277,279	0.43	0.49	0.00	0.00	1.00
Age	277,279	34.94	14.28	34.00	12.00	96.00
Friend taking Antidepressant	275,877	0.66	0.47	1.00	0.00	1.00

table S4.

Pearson Correlation between individual-level explanatory variables

	1.	2.	3.	4.	5.	6.
1. Spatial Diversity	1.00					
2. Local Cohesion	0.32	1.00				
3. Degree	-0.22	-0.55	1.00			
4. Friends within 50 km	-0.43	-0.12	-0.04	1.00		
5. Male	0.08	0.06	-0.12	-0.03	1.00	
6. Age	0.10	0.06	0.07	-0.06	-0.04	1.00
7. Friend taking Antidepressant	-0.15	-0.33	0.52	0.01	-0.10	0.19

table S5.

Probability of Antidepressant Use. Linear probability model (LPM) and logistic regression.

	LPM	*Placebo*	*Logistic*
	(1)	(2)	(3)
SD_i	−0.003***	0.001	−0.029**
	(0.000)	(0.000)	(0.014)
LC_i	−0.001***	0.000	−0.012
	(0.000)	(0.000)	(0.013)
ln (d_i)	−0.006***	0.000	−0.050***
	(0.000)	(0.001)	(0.014)
F^{50}	−0.001	−0.000	0.004
	(0.000)	(0.000)	(0.014)
Male_i	−0.020***	−0.000	−0.628***
	(0.001)	(0.001)	(0.023)
Age_i	0.026***	−0.000	0.688***
	(0.000)	(0.000)	(0.010)
Income_h	−0.001**	0.001	−0.049**
	(0.001)	(0.001)	(0.020)
Unemployment_h	0.002**	0.001	0.047**
	(0.001)	(0.001)	(0.023)
Constant	0.053***	0.039***	−3.067***
	(0.003)	(0.003)	(0.068)
County FE	Yes	Yes	Yes
N	265,719	265,719	265,719
R^2	0.022	0.000	

Note: Standard errors in parentheses. *p<0.1; **p<0.05; ***p<0.01

table S6

Robustness checks of Probability of Antidepressant Use estimation. Linear probability regressions. Standard errors in parentheses. *p<0.1; **p<0.05; ***p<0.01.

	Model 1	Model 2	Model 3	Model 4	Model 5	Model 6	Model 7
	Baseline	Strict Definition (ICD-10)	d≥ 10	Friend	Psychiatry	Borders	Low Education
SD_i	-0.0034***	-0.0034**	-0.0035***	-0.0033***	-0.0034***	-0.0034***	-0.0034***
	(0.000)	(0.000)	(0.000)	(0.000)	(0.000)	(0.000)	(0.000)
LC_i	-0.0012***	-0.0011**	-0.0016***	-0.0010**	-0.0012***	-0.0013***	-0.0012***
	(0.000)	(0.000)	(0.000)	(0.000)	(0.000)	(0.000)	(0.000)
ln (d_i)	-0.0055***	-0.0049***	-0.0056***	-0.0070***	-0.0055***	-0.0056***	-0.0055***
	(0.000)	(0.000)	(0.000)	(0.000)	(0.000)	(0.000)	(0.000)
F^{50}	0.0006	0.0002	-0.0007	-0.0007	-0.0006	-0.0007	-0.0006
	(0.000)	(0.000)	(0.000)	(0.000)	(0.000)	(0.000)	(0.000)
Male	-0.0201***	-0.0208***	-0.0200***	-0.0199***	-0.0201***	-0.0201***	-0.0201***
	(0.000)	(0.000)	(0.000)	(0.000)	(0.000)	(0.000)	(0.000)
Age	0.0261***	0.0245***	0.0260***	0.0255***	0.0261***	0.0261***	0.0261***
	(0.000)	(0.000)	(0.000)	(0.000)	(0.000)	(0.000)	(0.000)
$Unemployment_h$	0.0017***	0.0019**	0.0017**	0.0017**	0.0017**	0.0016**	0.0021**
	(0.000)	(0.000)	(0.000)	(0.000)	(0.000)	(0.000)	(0.000)
$Income_h$	-0.0014**	-0.0009	-0.0013**	-0.0011*	-0.0013**	-0.0016**	-0.0024***
	(0.000)	(0.000)	(0.000)	(0.000)	(0.000)	(0.000)	(0.000)
Friend takes Antidep.				0.0064***			
				(0.000)			
Distance to Psych					0.0000		
					(0.000)		
Km to any border						-0.0000**	
						(0.000)	
Low education							-0.0001*
							(0.000)
Constant	0.0517***	0.0488***	0.0519***	0.0476***	0.0515***	-0.0529***	0.0581***
	(0.002)	(0.002)	(0.002)	(0.002)	(0.002)	(0.000)	(0.004)
County FE	Yes	Yes	Yes	Yes	Yes	Yes	Yes
R^2	0.022	0.021	0.022	0.022	0.022	0.022	0.022
N	265719	265719	263894	265719	265719	265719	265719

table S7

Robustness checks of Probability of Antidepressant Use estimation. Logistic regressions. Standard errors in parentheses. *p<0.1; **p<0.05; ***p<0.01.

	Model 1	Model 2	Model 3	Model 4	Model 5	Model 6	Model7
	Baseline	Strict Definition (ICD-10)	d ≥ 10	Friend	Psychiatry	Borders	Low Education
SD_i	-0.0291**	-0.0353**	-0.0298**	-0.0256*	-0.0293**	-0.0308**	-0.0291**
	(0.014)	(0.014)	(0.014)	(0.014)	(0.014)	(0.014)	(0.014)
LC_i	-0.0117	-0.0095	-0.0252*	-0.0000	-0.0116	-0.0123	-0.0117
	(0.012)	(0.012)	(0.014)	(0.012)	(0.012)	(0.012)	(0.012)
ln (d_i)	-0.0432***	-0.0503***	-0.0527***	-0.1068***	-0.0501***	-0.0508***	-0.0497***
	(0.013)	(0.014)	(0.014)	(0.016)	(0.015)	(0.013)	(0.013)
F^{50}	0.0044	0.0160	0.0022	0.0024	0.0047	0.0026	0.0051
	(0.014)	(0.014)	(0.014)	(0.014)	(0.014)	(0.014)	(0.014)
Male	-0.6278***	-0.6884***	-0.6253***	-0.6200***	-0.6278***	-0.6279***	-0.6279***
	(0.023)	(0.000)	(0.023)	(0.023)	(0.023)	(0.023)	(0.023)
Age	0.6875***	0.6803***	0.6880***	0.6711***	0.6876***	0.6872***	0.6870***
	(0.010)	(0.000)	(0.010)	(0.010)	(0.010)	(0.010)	(0.023)
Unemployment$_h$	0.0553**	0.0019**	0.0477**	0.0480**	0.0468**	0.0442**	0.0553**
	(0.022)	(0.023)	(0.022)	(0.022)	(0.022)	(0.022)	(0.023)
Income$_h$	-0.0494**	-0.0367*	-0.0463**	-0.0398**	-0.04845**	-0.0530***	-0.0731***
	(0.019)	(0.020)	(0.019)	(0.019)	(0.019)	(0.019)	(0.026)
Friend takes Antidep.				0.2637***			
				(0.029)			
Distance to Psych					0.0003		
					(0.000)		
Km to any border						-0.0006*	
						(0.000)	
Low Education							-0.0041
							(0.002)
Constant	-3.1216***	-3.1872***	-3.1192***	-3.3019***	-3.1269***	-3.1013***	-2.9730***
	(0.056)	(0.058)	(0.056)	(0.060)	(0.057)	(0.057)	(0.121)
County FE	Yes	Yes	Yes	Yes	Yes	Yes	Yes
N	265719	265719	263894	265719	265719	265719	265719

table S8

Spatial separation of network variables on the probability of Antidepressant Use estimation. Linear probability regressions. EC_i denotes normalized clustering coefficient outside the hometown. $SD_i^{<20km}$ is spatial diversity of links to settlements that are less than 20kms away. $SD_i^{>20km}$ is spatial diversity of links to settlements that are more than 20kms away.

	(1)	(2)	(3)	(4)	(5)
LC_i	-0.002**				-0.002***
	(0.000)				(0.001)
EC_i		0.001			0.001
		(0.001)			(0.000)
$SD_i^{<20km}$			-0.003***		-0.002***
			(0.000)		(0.001)
$SD_i^{>20km}$				0.001	0.000
				(0.000)	(0.000)
$ln(d_i)$	-0.01***	-0.002***	-0.005***	-0.004***	-0.004***
	(0.000)	(0.001)	(0.000)	(0.000)	(0.000)
F^{50}_i	0.001**	0.001**	0.001***	0.001***	0.001
	(0.000)	(0.000)	(0.000)	(0.000)	(0.000)
Constant	0.051***	0.052***	0.051***	0.052***	0.051***
	(0.002)	(0.002)	(0.002)	(0.002)	(0.003)
Controls	Yes	Yes	Yes	Yes	Yes
County FE	Yes	Yes	Yes	Yes	Yes
R^2	0.021	0.022	0.022	0.022	0.023
N	265719	257265	234651	232045	214822

Note: Standard errors in parentheses. *p<0.1; **p<0.05; ***p<0.01

table S9.

Probability of Antidepressant Use with interaction terms. Linear probability regression.

	Erdős-Rényi normalized LC	*Degree-normalized LC*	*Non-normalized LC*
	(1)	(2)	(3)
SD_i	-0.004^{***}	-0.003***	-0.003^{***}
	(0.000)	(0.000)	(0.000)
LC_i	-0.002^{***}	−0.002***	-0.001^{***}
	(0.000)	(0.000)	(0.000)
$SD_i \times LC_i$	0.001***	0.001***	0.000
	(0.000)	(0.000)	(0.000)
$\ln(d_i)$	-0.006^{***}	−0.005***	-0.006^{***}
	(0.000)	(0.000)	(0.000)
$F50$	−0.001	−0.000	−0.000
	(0.000)	(0.000)	(0.000)
Male_i	-0.020^{***}	−0.020***	-0.020^{***}
	(0.001)	(0.001)	(0.001)
Age_i	0.026^{***}	0.026***	0.026^{***}
	(0.000)	(0.000)	(0.000)
Income_h	-0.001^{**}	−0.002**	-0.002^{**}
	(0.001)	(0.001)	(0.001)
Unemployment_h	0.002^{**}	0.002**	0.002^{**}
	(0.001)	(0.001)	(0.001)
Constant	0.053^{***}	0.053***	0.053^{***}
	(0.003)	(0.003)	(0.003)
County FE	Yes	Yes	Yes
N	265,719	272,480	272,480
R^2	0.022	0.022	0.022

Note: Standard errors in parentheses. *p<0.1; **p<0.05; ***p<0.01

table S10

Antidepressant use, distance to psychiatric centers and usage rate in the region

	Probability of Antidepressant Use		
	(1)	(2)	(3)
Distance to psychiatric centers (km)	0.0005		0.0002
	(0.001)		(0.001)
Antidepressant usage rate in the region (log)		0.892***	0.891***
		(0.054)	(0.054)
Constant	−3.272***	−0.417**	−0.426**
	(0.022)	(0.173)	(0.175)
Observations	277,344	277,344	277,344

Note: Standard errors in parentheses. *p<0.1; **p<0.05; ***p<0.01

table S11

Inclusion of predicted probability of antidepressant use as control variable

	Probability of Antidepressant	Days of Therapy in 2011	
		All obs	Antidepressant Users
	(1)	(2)	(3)
SD_i	−0.003***	−0.018***	−0.026*
	(0.000)	(0.002)	(0.015)
LC_i	−0.001**	−0.006**	−0.000
	(0.000)	(0.002)	(0.013)
ln(d_i)	−0.005***	−0.029***	−0.016
	(0.000)	(0.002)	(0.014)
$F^5 0$	−0.000	−0.003	−0.016
	(0.000)	(0.002)	(0.014)
Male$_i$	-0.020***	−0.103***	−0.095***
	(0.001)	(0.004)	(0.024)
Age$_i$	0.026***	0.133***	0.168***
	(0.000)	(0.002)	(0.013)
Income$_h$	−0.001**	−0.007**	−0.017
	(0.001)	(0.003)	(0.020)
Unemployment$_h$	0.001	0.004	−0.013
	(0.001)	(0.004)	(0.023)
$\hat{A}_h$	1.100***	5.539***	1.939
	(0.091)	(0.456)	(2.436)
Constant	0.011**	0.053**	4.852***
	(0.004)	(0.022)	(0.116)
Observations	265,719	265,719	9,769
R^2	0.023	0.024	0.028

Note: Standard errors in parentheses. *p<0.1; **p<0.05; ***p<0.01

table S12.

Days of Therapy (DOT) in subsequent years. Linear regression controlling for DOT in 2011.

	2012	2013	2014	2015
	(1)	(2)	(3)	(4)
Antidepressant DOT 2011	1.498***	1.393***	1.300***	1.222***
	(0.018)	(0.020)	(0.021)	(0.022)
Spatial Diversity	−0.054**	−0.071**	−0.075**	−0.071**
	(0.027)	(0.031)	(0.033)	(0.035)
Local Cohesion	−0.009	−0.023	−0.012	−0.019
	(0.024)	(0.027)	(0.031)	(0.032)
Degree	−0.013	−0.057*	−0.063**	−0.043
	(0.026)	(0.030)	(0.031)	(0.033)
Friends within 50 km	0.032	−0.014	−0.020	−0.036
	(0.026)	(0.030)	(0.031)	(0.032)
Male	−0.210***	−0.247***	−0.316***	−0.302***
	(0.044)	(0.050)	(0.052)	(0.054)
Age	0.217***	0.334***	0.359***	0.312***
	(0.023)	(0.027)	(0.028)	(0.029)
Income per capita	−0.023	0.025	0.062	−0.002
	(0.037)	(0.043)	(0.044)	(0.045)
Unemployment rate	−0.010	0.021	0.058	−0.015
	(0.043)	(0.049)	(0.051)	(0.053)
Constant	−4.152***	−4.064***	−3.618***	−3.250***
	(0.153)	(0.176)	(0.182)	(0.188)
Observations	9,769	9,769	9,769	9,769
R^2	0.447	0.355	0.317	0.276
Adjusted R^2	0.445	0.353	0.315	0.274

Note: Standard errors in parentheses. *p<0.1; **p<0.05; ***p<0.01

table S13.

Delta Days of Therapy (DOT, $Z_{i,t}$) in subsequent years. Linear regression controlling for DOT in 2011.

	$\Delta Z_{i,2012}$	$\Delta Z_{i,2013}$	$\Delta Z_{i,2014}$	$\Delta Z_{i,2015}$
	(1)	(2)	(3)	(4)
Antidepressant DOT 2011	0.498^{***}	0.393^{***}	0.300^{***}	0.222^{***}
	(0.018)	(0.020)	(0.021)	(0.022)
Spatial Diversity	-0.054^{**}	-0.071^{**}	-0.071^{hh}	-0.070^{**}
	(0.027)	(0.031)	(0.032)	(0.033)
Local Cohesion	−0.009	−0.023	−0.005	−0.019
	(0.024)	(0.027)	(0.028)	(0.029)
Degree	−0.013	-0.057^{*}	-0.061^{**}	−0.043
	(0.026)	(0.030)	(0.031)	(0.032)
Friends within 50 km	0.032	−0.014	−0.019	−0.036
	(0.026)	(0.030)	(0.031)	(0.032)
Male	-0.210^{***}	-0.247^{***}	-0.317^{***}	-0.302^{***}
	(0.044)	(0.050)	(0.052)	(0.053)
Age	0.217^{***}	0.334^{***}	0.359^{***}	0.312^{***}
	(0.023)	(0.027)	(0.028)	(0.029)
Income per capita	−0.023	0.025	0.061	−0.002
	(0.037)	(0.043)	(0.044)	(0.045)
Unemployment rate	−0.010	0.021	0.058	−0.015
	(0.043)	(0.049)	(0.051)	(0.053)
Constant	-4.152^{***}	-4.064^{***}	-3.616^{***}	-3.250^{***}
	(0.153)	(0.176)	(0.182)	(0.188)
Observations	9,769	9,769	9,769	9,769
R^2	0.102	0.069	0.055	0.036
Adjusted R^2	0.100	0.066	0.052	0.033

Note: Standard errors in parentheses. $^{*}p<0.1$; $^{**}p<0.05$; $^{***}p<0.01$

table S14.

Delta Days of Therapy (DOT, $Z_{i,t}$) in subsequent years. Linear regression controlling for DOT in 2011 and for Antidepressant Access.

	DOT 2012	DOT 2013	DOT 2014	DOT 2015
DOT 2011	1.497***	1.392***	1.299***	1.221***
	(0.018)	(0.020)	(0.021)	(0.022)
Antidepressant Access	4.829	9.443^{h}	11.284**	15.183***
	(4.482)	(5.165)	(5.325)	(5.505)
SD_i	−0.104***	−0.125***	−0.147***	−0.140***
	(0.036)	(0.041)	(0.042)	(0.044)
LC_i	−0.011	−0.025	−0.008	−0.020
	(0.024)	(0.027)	(0.028)	(0.029)
$ln(d_i)$	−0.011	−0.052*	−0.057*	−0.038
	(0.026)	(0.030)	(0.031)	(0.032)
F_{50}	0.035	−0.009	−0.014	−0.030
	(0.026)	(0.030)	(0.031)	(0.032)
Male_i	−0.213***	−0.249***	−0.321***	−0.306***
	(0.044)	(0.050)	(0.052)	(0.053)
Age_i	0.218***	0.335***	0.360***	0.313***
	(0.023)	(0.027)	(0.028)	(0.029)
Income_h	−0.027	0.019	0.055	−0.011
	(0.037)	(0.043)	(0.044)	(0.045)
Unemployment_h	−0.019	0.007	0.041	−0.038
	(0.043)	(0.050)	(0.051)	(0.053)
$SD_i \times \text{Male}_i$	0.058	0.010	0.093^{h}	0.085
	(0.046)	(0.053)	(0.055)	(0.056)
$SD_i \times \text{Age}_i$	0.048**	0.074***	0.073**	0.067**
	(0.024)	(0.028)	(0.029)	(0.030)
$SD_i \times \text{Income}_h$	−0.029	−0.048^{h}	−0.027	−0.036
	(0.023)	(0.026)	(0.027)	(0.028)
Constant	−4.326***	−4.410***	−4.034***	−3.817***
	(0.230)	(0.265)	(0.273)	(0.282)
Observations	9,769	9,769	9,769	9,769
R^2	0.447	0.355	0.318	0.277
Adjusted R^2	0.445	0.353	0.316	0.274

Note: Standard errors in parentheses. *p<0.1; **p<0.05; ***p<0.01

table S15.

Regressions by dropping individuals reporting a birthday in the first month of the year and first day of the month.

	Probability of Antidepressants	*Days of Treatment, 2013*
	(1)	(2)
DOT_{2011}		1.420***
		(0.025)
SD_i	−0.003***	-0.083**
	(0.001)	(0.038)
LC_i	−0.001***	−0.025
	(0.001)	(0.034)
$ln\ (d_i)$	−0.006***	−0.072**
	(0.001)	(0.037)
$F\ 50$	−0.001	−0.007
	(0.001)	(0.037)
$Male_i$	−0.019***	−0.244***
	(0.001)	(0.061)
Age_i	0.026***	0.314***
	(0.000)	(0.033)
Access to Antidep		16.102**
		(6.586)
Constant	0.053***	-4.839***
	(0.004)	(0.359)
County FE	Yes	Yes
N	170,776	6,322
R^2	0.022	0.363
